# Two-Dimensional Electronic Spectroscopy of Rhodamine 700 Using an 8 fs Ultrabroadband Laser Source and Full-Wavelength Reference Detection

**DOI:** 10.1021/acs.jpca.4c08494

**Published:** 2025-03-05

**Authors:** Camilla Gajo, Caleb J. C. Jordan, Thomas A. A. Oliver

**Affiliations:** School of Chemistry, University of Bristol, Bristol BS8 1TS, U.K.

## Abstract

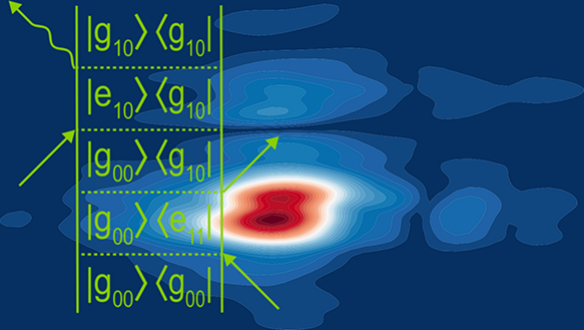

Two-dimensional electronic spectroscopy (2DES) is one of the premier tools for investigating photoinduced condensed phase dynamics, combining high temporal and spectral resolution to probe ultrafast phenomena. We have coupled an ultrabroadband laser source generated with a hollow-core fiber, compressing pulses to have a pulse duration of 8 fs, with a boxcars 2DES interferometer constructed from only conventional optics. The resulting ultrabroad bandwidth and high temporal resolution allow for superior spectral coverage of the typically broad molecular line shapes in the near-IR/visible region in room temperature solutions, and the exploration of the excited state dynamics at the earliest time epoch in complex systems. The new spectrometer is characterized by examining the dynamics of the dye molecule Rhodamine 700 in methanol solution. These data exhibit rich vibrational wavepacket dynamics, with 2DES data unraveling key molecular vibronic couplings between multiple vibrational modes. For the first time in a degenerate broadband 2DES experiment, we demonstrate the implementation of full-wavelength reference detection to correct wavelength-dependent laser intensity fluctuations. The net result is a 4–5× increased signal-to-noise (S/N) ratio compared to data acquired without reference detection, yielding a typical S/N ratio = 28. The increased S/N ratio facilitates more rapid data acquisition and examination of samples at lower optical densities, and thus concentrations, than typically used in 2DES experiments. These advances will help to alleviate the typical high demands on precious samples in 2DES measurements.

## 1 Introduction

Two-dimensional electronic spectroscopy (2DES) is one of the most incisive experimental methods to explore ultrafast excited state condensed phase dynamics of molecules,^1−4^ molecular aggregates,^5−7^ photoactive proteins,^8,9^ and nanomaterials.^10−14^ To date, this multidimensional spectroscopic technique has revealed a wealth of dynamical information about ultrafast electronic energy transfer,^5−7,15−19^ photoinduced charge transfer,^13,20−22^ and nonadiabatic dynamics.^1,8,12,23−27^ 2DES is uniquely disposed to unravel these phenomena due to its ability to correlate excitation and detection frequencies as a function of ultrafast system evolution. Further, the incisive technique allows for the discrimination between overlapping transient features, identification of cross-peaks, elucidation of coherences between states, resolution of homogeneous and inhomogeneous line shape components, and deduction of spectral diffusion time scales arising from system–bath coupling.^28−35^

As ultrafast lasers have become more technologically advanced, excitation sources for coherent multidimensional spectroscopies have achieved greater levels of sophistication, with experiments now incorporating laser sources that can fully cover broad molecular and supramolecular line shapes in solution between the visible and near-IR parts of the electromagnetic spectrum. The broader laser bandwidths accommodate shorter possible pulse durations, and as a result, the associated instrument response of spectrometers has also improved, allowing access to the very earliest events of excited state evolution. To realize these ultrashort ultrabroadband light sources, several research groups have moved away from more conventional laser sources such as noncollinear optical parametric amplifiers and taken advantage of advances made by the ultrafast X-ray community to generate single or subcycle visible/near-IR laser pulses required as a precursor to high-harmonic generation.^36,37^ These technologies are based on filamentation and self-phase modulation in pressurized gas cells or gas-filled hollow-core fibers to generate broadband supercontinua, which have been utilized as the laser source in several 2DES experiments.^38−47^ Alternatively, 2DES experiments using white light continuum sources have relied on generating the laser pulses in a more conventional manner, e.g., filamentation in a bulk media such as yttrium aluminum garnet crystals.^48,49^ This latter approach yields sources with far lower pulse energies but nonetheless has sufficient peak power intensity for nonlinear multidimensional spectroscopy measurements.

To remove the effects of laser noise on transient absorption data, shot-to-shot normalization of the wavelength-dependent fluctuations in the white light supercontinuum probe vastly improves the technique’s sensitivity.^50−53^ This is realized by the simultaneous acquisition of the signal/probe and a second reference beam derived from the same laser source on a shot-to-shot basis. Despite the demanding signal-to-noise ratios required to measure often weak coherent dynamic signatures in 2DES data, to date, no 2DES experiment using *degenerate* broadband excitation sources has implemented full shot-to-shot correction for laser instabilities as a function of the entire laser bandwidth. Prior studies have corrected for shot-to-shot instabilities either with a single-element photodiode^54,55^ and thus have not accounted for the wavelength-dependent laser instability or have only done so in two-color 2DES experiments with visible pump bandwidths of ∼50 nm and a separate broader bandwidth white light supercontinuum probe.^56,57^

We detail the innovation of a 2DES spectrometer using a 1 kHz repetition rate ultrabroadband white light continuum laser source, with an 8 fs instrument response and full-wavelength reference detection. Our spectrometer is novel because it is the first to incorporate the following multiple important design aspects: (i) the interferometer is realized in the boxcars geometry yielding background free signals, and resultantly an improved by an order-of-magnitude S/N ratio compared to 2DES data acquired in the pump–probe geometry;^58−60^ (ii) taking inspiration from prior designs,^55,61,62^ the phase-stable spectrometer was constructed entirely from conventional optics such as beam splitters and utilized pairwise beam manipulation, which results in the data being acquired in the fully rotated frame; (iii) the 2DES experiment is driven by a white light supercontinuum (spanning 460–1030 nm) generated in a hollow-core fiber laser source which is compressed to 8 fs, and is therefore capable of probing very early-time dynamics in systems with broad molecular absorption line shapes throughout the visible and near-IR spectral regions; and (iv) full-wavelength reference detection is implemented to correct for wavelength-dependent laser intensity fluctuations which has a net result on increasing the S/N ratio by 4–5 times, and the overall sensitivity of the experiment (with a typical S/N = 28). Combined, this overall approach yields 2DES spectra at single waiting times within one minute using a 1 kHz repetition rate laser and generating the required time delays with mechanical delay stages. We demonstrate the capabilities of the new spectrometer by studying the coherent wavepacket dynamics of Rhodamine 700 (Rhod700), revealing rich molecular vibronic coupling between multiple vibrational modes. These studies were conducted at typical optical densities used for nonlinear spectroscopic measurements and with more dilute samples (OD = 0.1) of ∼55 μM concentration, demonstrating the enhanced sensitivity wavelength-dependent reference detection affords 2DES.

## 2 Experimental and Computational Methods

### 2.1 White Light Generation and Pulse Compression

The output of a commercial amplified ultrafast laser system (Coherent, Astrella, 5 mJ, 1 kHz, 35 fs) was used to generate a white light continuum (WLC) from a hollow-core fiber (Imperial Consultants). The output laser beam pointing was actively stabilized with a commercial steering correction system (Newport, Guidestar II), achieving a target stability of 5 μm over 3.5 m. A portion of the laser output was attenuated to 400 μJ using a λ/2 waveplate–polarizer pair (Eksma 461–4215 and 420–1506E, respectively) and focused into a 1 m long, 250 μm diameter hollow-core fiber with an *f* = 1000 mm silver-coated concave mirror. The fiber was filled with a static pressure of ∼1.2 bar Argon (N5.5 Research grade, BOC), generating a supercontinuum spanning more than one octave: 460–1030 nm (see Figure S1). The beam diameter was measured to be 160 ± 10 μm at the fiber entrance and fine-tuned with an iris to optimize the WLC output spectrum and power stability. The broadband output was collimated with an *f* = 1000 mm concave silver mirror, and the beam diameter was reduced to ∼3 mm by using a reflective 3:2 telescope. Near-infrared wavelengths of >750 nm generated by the fiber were attenuated using an absorptive glass filter (Schott, KG5). The filtered output of the fiber had a 0.3% shot-to-shot power stability. The second-order group-velocity dispersion of the WLC was compensated for with two pairs of double-angled chirped mirrors (Ultrafast Innovations, PC70) and fine-tuned with a pair of fused-silica wedges (Newport, 23RQ12–02-M). The chirped mirrors transmitted light at wavelengths <500 nm. The precise number of reflections on chirped mirrors and the amount of inserted glass required to minimize the pulse duration was determined by iterative *in situ* transient-grating frequency-resolved optical gating (TG-FROG^63^) measurements of pure methanol in the sample flow cell under identical experimental conditions used for 2DES experiments.

### 2.2 2DES Spectrometer

**Figure 1:**
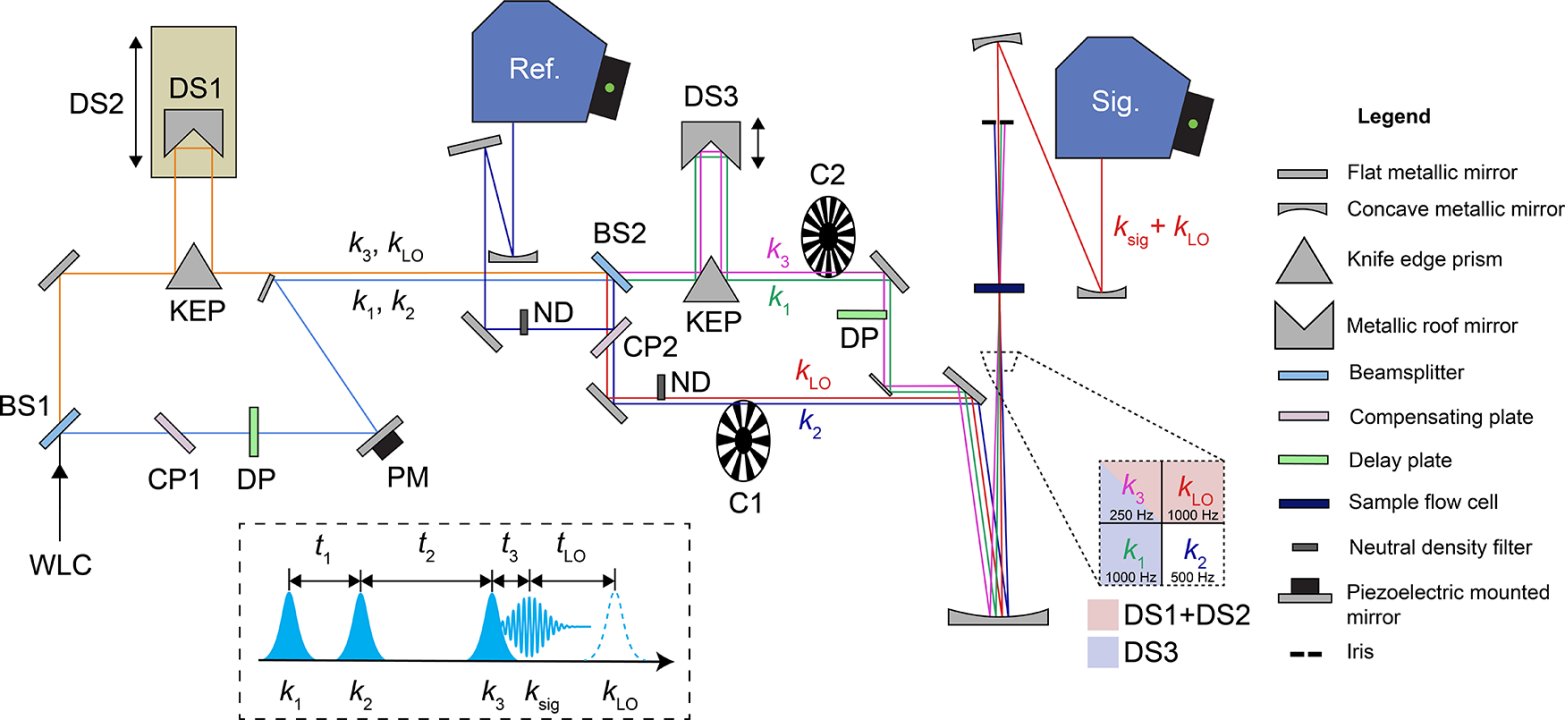
Schematic optical layout of the 2DES interferometer. Laser beams in the spectrometer are vertically offset at BS2; however, in the diagram, this is illustrated as a horizontal offset to facilitate easier tracing of laser paths. The inset dashed box contains the 2DES laser pulse sequence and definition of pulses and interpulse time delays.

In our mixed time-frequency domain 2DES experiment,^64^ we define our labels for the interpulse time delays and χ^3^ pulse sequence in the following section. With reference to Figure 1, the pump pulses that excite the sample are labeled by their associated *k*-vectors *k*_1_ and *k*_2_, and the interpulse coherence time delay, *t*_1_. The sample subsequently interacts with *k*_3_, which probes the system’s state at the waiting time delay, *t*_2_. This pulse sequence results in signal emission, *k*_sig_, at echo time, *t*_3_. The signal is heterodyne detected with an attenuated replica pulse or local oscillator (*k*_LO_), which amplifies the signal and facilitates the extraction of phase information.^2^ 2DES experiments in the boxcars geometry mean that both rephasing and nonrephasing phase-matched signals must be separately recorded to obtain a total 2DES spectrum and have signal wavevectors, ***k***_**sig**_ = −***k***_**1**_ + ***k***_**2**_ + ***k***_**3**_ and −***k***_**2**_ + ***k***_**1**_ + ***k***_**3**_, respectively.

The WLC laser output polarization and pulse intensity into the 2DES interferometer were controlled with an achromatic λ/2 waveplate (Thorlabs, SAHWP05M-700) and variable neutral density filter (NDC-100C-4). The 2DES optical setup is shown schematically in Figure 1. The first 50:50 beamsplitter in the spectrometer, BS1 (Layertec, 147970), creates a pair of broadband replica pulses. The transmitted beam, which pulses *k*_3_ and *k*_LO_ derive from, was directed toward a second identically coated beamsplitter with different thicknesses, BS2 (Layertec, 106896), but routed via a roof mirror (PLX, RM-101–1E) mounted on the first of two piezoelectric delay stages (DS1). The piezoelectric stages (Physik Instrumente, P-622Z.CD stages, and E727 controller) have a positioning accuracy of <15 nm, corresponding to a time delay of <0.1 fs. The DS1 piezoelectric stage was mounted on a long-travel ball screw delay stage (DS2, Physik Instrumente, M-531.DG1 controlled by C-863.11). The reflected arm generated at BS1 (precursor to *k*_1_ and *k*_2_) was delayed by the same distance and directed toward BS2. One mirror on this delay line was mounted on a piezoelectric diaphragm element (PM), and its purpose is explained later. The two beams generated by BS1 are vertically offset along the two different paths and, when recombined at BS2, are displaced so they do not spatially overlap. BS2 creates the four beams required for boxcar 2DES measurements, with the transmitted pair of beams (*k*_1_ and *k*_3_) directed to a second roof mirror mounted on a second piezoelectric state (DS3). The reflected arm of the beamsplitter generates *k*_2_ and *k*_LO_ pulses.

Compensating plates made from substrates identical to those of the beam splitters were inserted into both arms of the spectrometer to ensure that all beams had the same group-velocity dispersion. The angles of the plates were fine-tuned by diffracting pulse pairs through a 30 μm diameter pinhole at the sample position and measuring the wavelength-dependent interference pattern as a function of the interpulse time delay. This procedure was iterated until the central fringe in the interference pattern displayed no tilt.

The three delay stages were used to precisely manipulate the timing of the three pulses, the local oscillator, and their phase-matched interactions with the sample to generate the 2DES signals. Positive *t*_2_ delays were created by delaying *k*_3_ and *k*_LO_ relative to *k*_1_ and *k*_2_ and achieved by increasing the path length controlled by the DS2 (ball screw) stage. To create the *t*_1_ time delay, DS3 or a combination of DS1 and DS3 stage movements were necessary to generate nonrephasing and rephasing signals, respectively, as previously described.^55,61,62^ To acquire nonrephasing signals (−*t*_1_) at a fixed waiting time, *k*_1_ and *k*_3_ are delayed by lengthening the path traveled on DS3, which results in *k*_2_ interacting with the sample first. This also linearly decreases the time delay between the local oscillator and the *k*_3_ probe pulse with increasing *t*_1_ delay. To generate the rephasing signal (+*t*_1_), *k*_1_ must excite the sample first, and thus, the beam path controlled by DS3 is shortened. However, as DS3 controls the timing of both *k*_1_ and *k*_3_ pulses, they both arrive at the sample earlier, which resultantly also decreases the *t*_2_ delay. To preserve a fixed value of the waiting time during the rephasing signal acquisition, DS1 is incrementally scanned by the same distance as DS3, but in the opposite direction. Consequentially, the *k*_3_ and *k*_LO_ time delay increase as a function of *t*_1_. The advantage of the local oscillator being scanned in time as a function of the *t*_1_ delay is that the signal is acquired in the fully rotating frame.^55,61,62^ Therefore, the requirements to sample the *t*_1_ delay and ensure that the Nyquist sampling theorem is satisfied are greatly reduced. This approach also lowers the typically stringent phase stability requirement in the *t*_1_ delay.^65^ The time delays created by the DS1 and DS3 stages were verified by spectral interferometric measurements.

Using the boxcar geometry, the four beams were focused into the sample using a concave silver mirror (*f =* 200 mm) with a crossing angle of 4°. The spatial overlap of all four beams was verified using a beam profiling camera. Time zero between *k*_1_, *k*_2_, and *k*_3_ was found by iteratively maximizing the intensity of the TG-FROG response of a solvent-only sample. The pulse energy associated with each *k*_1_, *k*_2_, and *k*_3_ beam was 5 nJ at the sample position in a spot size of 50 μm (fwhm). The local oscillator was attenuated by a 1 mm thick BK7 ND 2 filter, which also delayed the LO by ∼1900 fs relative to the *k*_3_ probe pulse. To ensure that the fringes in the spectral interferogram were sufficiently resolved, the relative delay of the LO to the *k*_3_ pulse was reduced to ∼280 fs by the insertion of 1 mm thick fused-silica delay plates, DP, into the *k*_1_, *k*_2_, and *k*_3_ paths.

The 2DES signal and collinear local oscillator were collected, collimated (*f* = 250 mm), and focused (*f* = 150 mm) into a spectrograph (Andor, Shamrock 163) that wavelength disperses the signal and local oscillator onto a linear 1024-element CCD (S7030-1006, Hamamatsu) array detector (Entwicklungsbüro EB Stresing). This detection method acts as a Fourier transform operation, converting *t*_3_ into its conjugate ω_3_. The resulting interferograms were digitized and recorded using Labview (National Instruments). For every *t*_2_ delay, ω_3_ interferograms are acquired as a function of the *t*_1_ delay generating a 2DES (*t*_1_–ω_3_) surface. These data are processed and subsequently Fourier-transformed along *t*_1_ to yield 2DES ω_1_–ω_3_(*t*_2_) correlation maps.

To remove scatter contributions from the heterodyne-detected 2DES signal, *k*_2_ and *k*_3_ were modulated with synchronized optical choppers (MC2000B, Thorlabs) at 500 and 250 kHz, respectively. The scattering contributions were subtracted as detailed previously.^62^ This significantly improved the quality of the recorded 2DES signals but also reduced the duty cycle of the experiment by 1/4.

For all 2DES measurements on Rhod700, the *t*_1_ coherence time was scanned from −60 to +60 fs in 1 fs steps, and the *t*_2_ waiting time was scanned from −96 to 1000 fs in 8 fs steps. Each reading at a given *t*_1_ delay comprised of 100 laser shots was acquired on a shot-to-shot basis (corresponding to 25 measurements) and was averaged over 3 cycles. The laser beams were all *s*-polarized, and thus, the data were collected in a parallel polarization configuration that generally maximizes the coherent wavepacket signal present in monomeric systems within the first few picoseconds.

Degenerate pump–probe data were also acquired in the same 2DES interferometer using *k*_2_ and *k*_LO_ beams. The pump–probe measurement was used to determine how the waiting time in 2DES data acquisition should be sampled based on the associated wavepacket periods and also for phasing of the 2DES data using the projection-slice theorem.^2^ These data comprised 500 shots per *t*_2_ delay and were averaged for 10 cycles. Pump–probe data were contaminated by pump scatter, which was suppressed by modulating the *t*_2_ delay by a factor of π on alternative laser shots, as previously demonstrated for 2DES and 2DIR experiments acquired in the pump–probe geometry.^66,67^ This was realized by mounting a mirror on the *k*_2_ path to a piezoelectric diaphragm element (RS Pro, 724–3162, labeled PM in Figure 1) and applying a sinewave (generated with an arbitrary waveform generator, Rigol DG822) to the mirror at a quarter of the repetition rate of the laser (250 Hz). The amplitude of the modulation applied to the piezoelectric element was tuned until the scatter was minimized.

### 2.3 Full-Wavelength Reference Detection

A replica spectrum (reference) was recorded on a shot-to-shot basis by directing a back reflection from a compensating plate (see Section 2.1) into a separate beam path and imaged into a second identical spectrograph and synchronized CCD detector. There are inherent problems with reference detection, such as uncorrelated noise of the two signal and reference detectors, and differences in imaging lead to improper wavelength-to-wavelength normalization. To circumvent these two issues, a correlation matrix can be constructed to ensure perfect pixel-to-pixel mapping and account for uncorrelated detector noise. These efforts have been pioneered in multidimensional spectroscopies by Ge and co-workers^68^ and implemented for different numbers of sequential pulse acquisitions by the groups of Cheatum^69^ and Meech.^57^ The full derivation is given in these prior reports, and our implementation is described here briefly: with all four beams present at the sample position, tens of thousands of laser shots were acquired simultaneously on both the signal and reference CCD detectors. During this data acquisition, zero signal intensity was ensured by moving the delay stages to sufficiently long *t*_1_ and *t*_2_ time delays. The reference shots were converted into a series of signal measurements from which a reference correlation matrix was determined, following the method of Ge and co-workers.^68^ The correlation matrix was then used to correct for wavelength-dependent laser fluctuations in real time, e.g., during data acquisition. For example, pump–probe (Δ*T*/*T*) data were acquired using the following relation for *N* laser shots
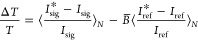
1where *I*_sig_ and *I*_ref_ are the wavelength-dependent spectral intensities on the signal and reference detectors for the same laser shot and *B̅* is the reference matrix. Typically, 20,000 reference shots were acquired for pump–probe measurements or 40,000 reference shots for 2DES measurements. For comparisons of data acquired with and without reference detection, the data were acquired simultaneously during the respective measurements.

### 2.4 Sample

Rhod700 perchlorate (Radiant Dyes GmbH) was dissolved in methanol (Merck, ≥99.9%), filtered with a syringe filter (0.2 μm), and diluted to have an absorbance of 0.3 or 0.1 at 645 nm in a flow cell with a 200 μm path length (Starna Scientific, 48/UTWA2/Q/0.2). The samples were continually flowed throughout the experiments using a peristaltic pump (Masterflex, MFLX07528–10). Steady-state UV–visible absorption spectra before and after the experiments showed no evidence of sample degradation.

### 2.5 Density Functional Theory Calculations

Density functional theory (DFT) and time-dependent DFT (TDDFT) were used to calculate the minimum energy geometries and associated vibrational frequencies of Rhod700 in the S_0_ and S_1_ electronic states. TDDFT calculations used the Tamm–Dancoff approximation (TDA). DFT and TDDFT calculations used the ωB97XD long-range corrected exchange–correlation functional, def2-SVP basis set, and an integral equation formalism polarizable continuum model (IEFPCM) for the methanol solvent. All calculations were performed in the Gaussian 16, revision C.01.^70^

## 3 Results and Discussion

### 3.1 Laser Pulse Characterization and 2DES Interferometer Phase Stability

**Figure 2:**
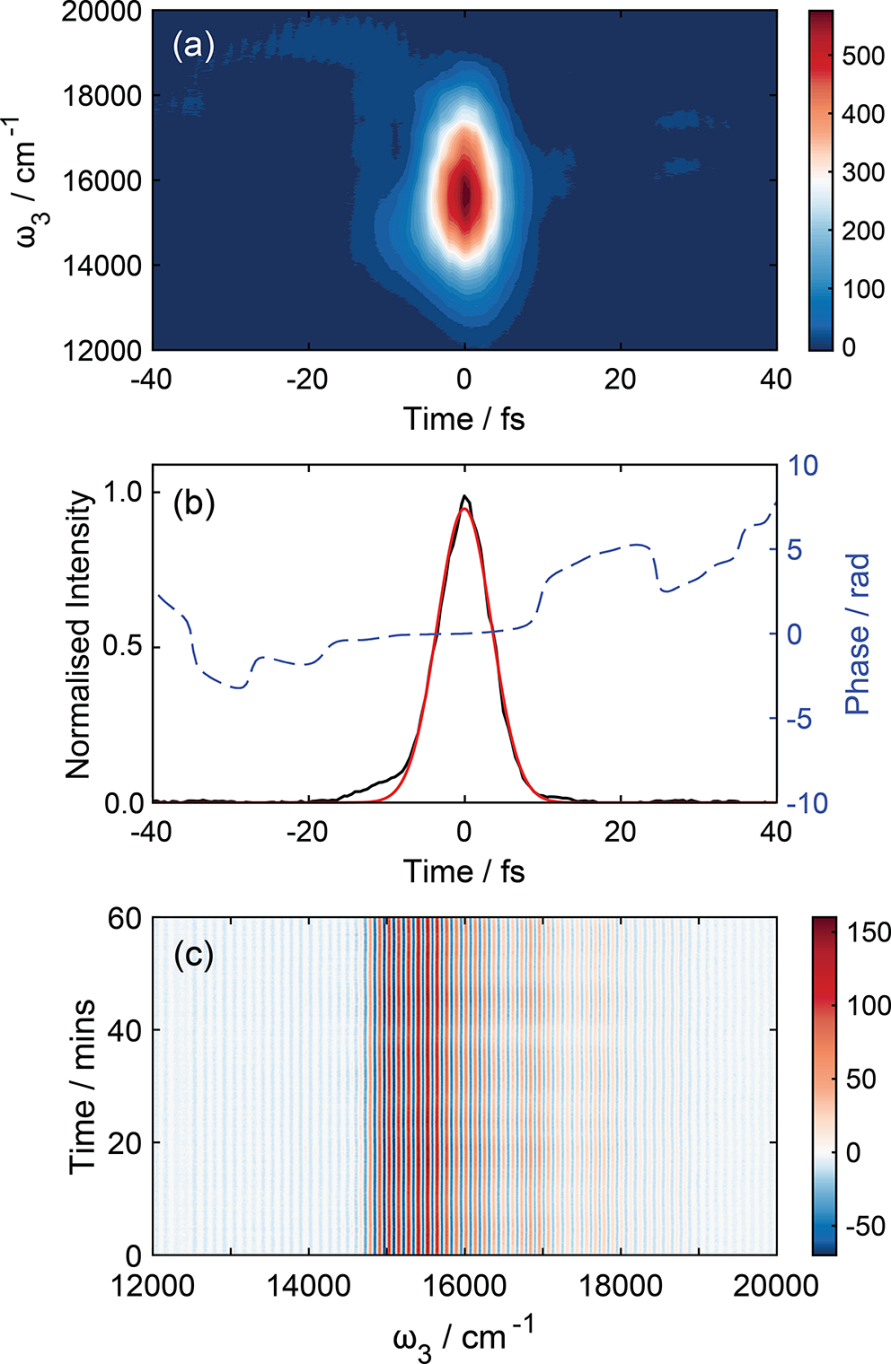
Characterization of laser pulse and 2DES spectrometer phase stability. (a) *In situ* TG-FROG pulse characterization of methanol in 200 μm path length flow cell at the sample position. (b) Integrated autocorrelation fit to a Gaussian function returning an 8 fs pulse duration and retrieved phase. (c) Interference fringes of the 2DES signal at *t*_1_ = 0 fs and *t*_2_ = 100 fs of a Rhod700 sample in methanol.

The instrument response of the 2DES spectrometer was determined by nonresonant TG-FROG measurements of methanol *in situ*. A typical TG-FROG trace acquired in a 200 μm path length flow cell is displayed in Figure 2a, with the integrated autocorrelation returned from this measurement and the retrieved phase given in Figure 2b. Fitting these data to a Gaussian function yields an 8 fs fwhm instrument response. There are no substantial wings or evident high-order group-velocity dispersion within these data. With the 61 THz (fwhm) laser bandwidth we have at the sample position, a time-bandwidth product of 0.488 is calculated, which is close to the transform limit. The TG-FROG traces were reproducible on a day-to-day basis with minimal or no changes to the beam pointing or compression required. With the broad signal bandwidth of Rhod700, the directional filter and phase-mismatch parameters^71^ were calculated to be 0.023 and 0.0037, respectively; both are within the acceptable bounds of ≪1.

The phase stability between pump pulse pairs, *k*_1_ and *k*_2_, was measured by acquiring the heterodyned detected 2DES signal of Rh700 in methanol at *t*_1_ = 0 fs and *t*_2_ = 100 fs. On time scales of over an hour (Figure 2c), a typical time frame that allows for 2DES data collection at ∼60 *t*_2_ delays, a phase stability of λ/70 at 510 nm was extracted from the interferogram.

The wavelength-dependent intensity fluctuations of our laser source are characterized in Figure S2. This analysis shows that the fluctuations are not uniform across the laser spectrum, demonstrating the need for wavelength-dependent intensity corrections as opposed to an integrated intensity correction, as previously implemented for 2DES.^54,55^

### 3.2 Rhodamine 700 in Methanol

#### 3.2.1 Pump–Probe Spectroscopy

**Figure 3:**
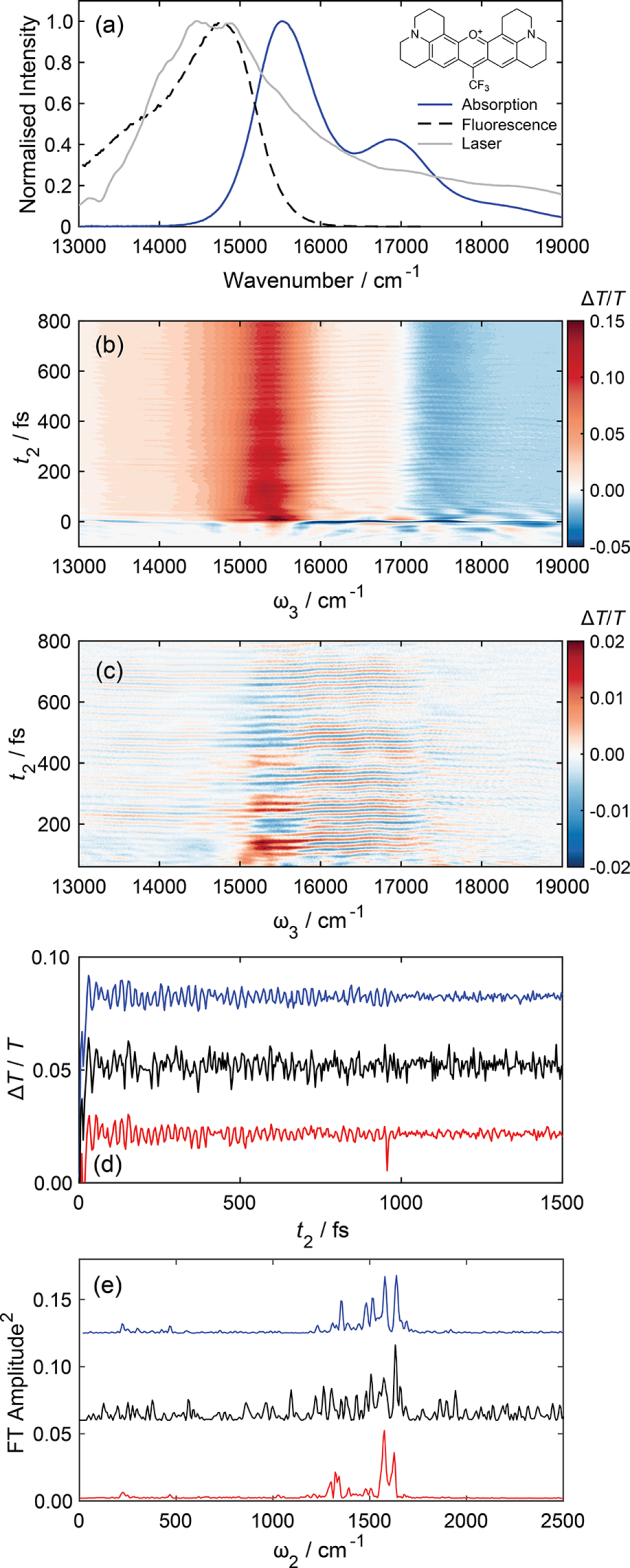
(a) Normalized steady-state absorption and fluorescence spectra of Rhodamine 700 in methanol solution. The laser spectrum at the sample position is also overlaid, with the dye’s chemical structure displayed inset. (b) Degenerate pump–probe data for waiting times between −100 and 800 fs. (c) Coherent wavepacket signals extracted from panel b. (d) Comparison of wavepacket dynamics at probe wavenumber 16,700 cm^–1^ for data acquired with (blue) and without (black) reference detection for 50 shots per *t*_2_ delay and 1 cycle, unreferenced data acquired with 500 shots (red). (e) Fourier transform along the *t*_2_ delay of data displayed in panel d highlights the signal-to-noise improvement achieved in pump–probe data by full-wavelength reference detection. For the purposes of comparison, the slices in panels d and e have been vertically offset.

The steady-state visible/near-IR absorption spectrum of Rhod700 in methanol (see Figure 3a) shows a clear vibrational progression, with the highest intensity peak centered at 15,530 cm^–1^ assigned to the S_1_ electronic origin. At higher wavenumbers, there is a major vibrational band at 16,920 cm^–1^, with a second less intense shoulder at 18,450 cm^–1^. The peak spacing between these three bands is ∼1500 cm^–1^. The fluorescence spectrum of Rhod700 in methanol peaks at 14,770 cm^–1^ exhibits a Stokes shift of 760 cm^–1^ and an appreciable amount of mirror symmetry with respect to the absorption spectrum. TDDFT/TDA/ωB97XD/def2-SVP calculations with an IEFPCM solvation model (Table S1) predicted the lowest energy excited (S_1_) state to be π* ← π in character with significant oscillator strength. The S_2_ state was found to be at significantly higher energies and predicted to have a far lower oscillator strength than the S_1_ transition—in agreement with steady-state absorption data acquired at shorter wavelengths (see Figure S4). Combined, these steady-state and computational data indicate that broadband visible (460–850 nm) photoexcitation of Rhod700 will populate only the S_1_ state and its associated manifold of vibrational levels.

Degenerate pump–probe spectra between −100 ≤ *t*_2_ ≤ 1600 fs are displayed in Figure 3b. These data were acquired by λ/2 modulation of the *t*_2_ delay, as described in Section 2.2, and a comparison of the data acquired without this scatter suppression method is shown in Figure S5. This alleviates the need for additional Fourier filtering along the probe dimension,^3^ or alternative heterodyne-detected transient grating measurements.^72,73^ Using the steady-state spectra to guide assignment, the dominant positive feature in the transient spectrum is attributed to overlapping ground state bleach (GSB) and stimulated emission (SE) signals. A less intense negative feature associated with S*_n_* ← S_1_ excited state absorption (ESA) is evident at higher probe wavenumbers. In these early-time pump–probe data, most of the signal amplitude originates from population relaxation dynamics. Festooned on top of the picosecond/nanosecond vibrational relaxation and excited state decay kinetics are high-frequency oscillatory signals from nuclear wavepackets associated with Franck–Condon active vibrational modes that are coupled to the S_1_–S_0_ electronic transition.

The probe wavelength-dependent wavepacket dynamics were isolated from the pump–probe spectra as previously described.^3,54,74,75^ Briefly, each probe wavelength was fitted to a smooth spline function to model the population relaxation dynamics and subtracted from the original data. The residuals correspond to the isolated coherent wavepacket signals (Figure 3c), which exhibit a strong probe wavenumber dependence and have multiple frequency components. The amplitude of the wavepacket decays to baseline within several picoseconds due to vibrational dephasing. A closer examination of the data reveals a node at ∼17,300 cm^–1^ corresponding to the intersection of the oppositely signed ESA and GSB/SE features. A second node is also evident at ∼15,200 cm^–1^ that coincides approximately with the crossing point between steady-state absorption and fluorescence spectra. As shown by Scholes, Cina, and co-workers,^34,54,75,76^ the node appears at energies that correspond to the minimum of the potential energy surface due to the interference of antiphased oscillations to the red or blue edges of the stimulated emission band. The time-dependent position of the node therefore tracks the time-evolving Stokes shift, which for a simple dye in methanol solution is expected to be multiphasic and dominated by a picosecond shifting time constant.^77^

Slices at single wavelengths of the isolated wavepacket dynamics at ω_3_ = 16,700 cm^–1^ (Figure 3d) show the vast S/N improvement achieved by our reference detection acquisition in the raw data collection for only 50 shots per *t*_2_ delay and 1 cycle. To achieve the same S/N ratio without referencing requires 500 shots per *t*_2_ delay and thus represents a factor of 10 times increase in data acquisition rate possible using our wavelength-dependent intensity correction method. Similar full-wavelength reference approaches have been demonstrated previously with transient absorption spectroscopy,^50,51^ yielding enhanced S/N ratios, but not with degenerate ultrabroadband 2DES spectroscopy. The impact of this improved signal-to-noise ratio is further illustrated in the Fourier transform of these wavepacket dynamics shown in Figure 3e, where the baseline of the unreferenced data acquired for the same number of laser shots is significantly more intense with a forest of peaks evident, which are clearly artifacts, than when compared to the referenced data.

**Figure 4:**
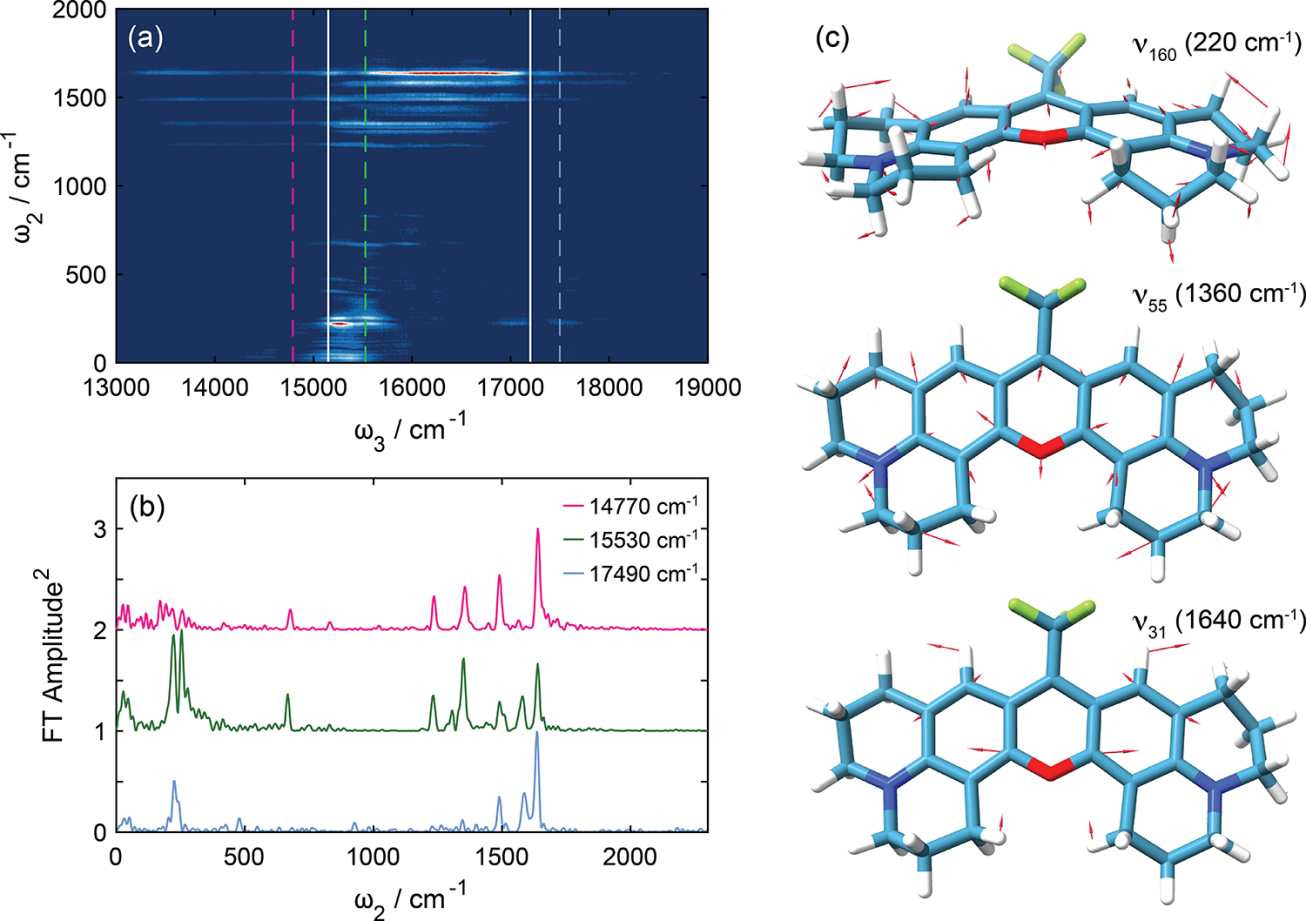
(a) Fourier-transform map of the coherent time-domain wavepackets (Figure 3c) as a function of the probe wavenumber. Green, pink, and blue dashed lines correspond to steady-state absorption, fluorescence, and ESA maxima, respectively. Solid white lines correspond to nodes formed between antiphased oscillations. (b) Fourier transform slices for probe wavenumbers associated with the maxima of GSB (15,530 cm^–1^), SE (14,770 cm^–1^), and ESA (17,490 cm^–1^) transients. Note that the data are vertically offset. (c) Three calculated Franck–Condon active normal modes of Rhod700, with overlaid arrows indicating major nuclear displacements. Further details on the mode assignment are given in Table S2.

Figure 4a shows the full wavepacket map for the pump–probe data recorded with reference detection with an evident probe wavenumber dependence on the vibrational wavepackets. Note that we report wavepacket frequencies in wavenumbers, but for the sake of simplicity, we use frequency to describe them. Slices in Figure 4b show the wavepacket frequencies associated with 14,770, 15,530, and 17,490 cm^–1^ anticipated to be dominated by GSB, ESA, and SE transients. Given there is significant overlap between these features and that vibrational frequencies associated with the same mode can often be similar on the ground and excited states, it is frequently unfeasible to ascribe wavepacket frequencies based on a simple correspondence with probe wavenumber. However, as shown previously, and we demonstrate for Rhod700, 2DES spectroscopy is capable of distinguishing between ground and excited state vibrational coherences,^24,78−84^ and thus, using 2DES measurements, we can assign the different wavepacket frequencies to coherent evolution on different electronic states.

The changes in the Rhod700 minimum energy geometry upon S_1_ ← S_0_ excitation were predicted using DFT and TDDFT calculations. Upon photoexcitation, the molecular framework of Rhod700 changes in two key ways: the central pyran aromatic ring expands with some modest changes evident in bond lengths within the flanking phenyl groups, as expected for a π* ← π transition in an aromatic molecule, Rhod700 significantly deviates away from planarity in the S_1_ state (152°) in contrast to the planar S_0_ minima (see Figure S3). Based on these results, significant Franck–Condon activity is anticipated in normal modes with nuclear displacements along these two coordinates, thus allowing us to propose assignments for the vibrational wavepacket frequencies.

The vibrational wavepacket normal mode assignments are given in Table S2 and can be divided into two subsets: ν_159_ and ν_160_ correspond to out-of-plane motions, and ν_31_, ν_47_, ν_55_, and ν_72_ to in-plane ring breathing modes. Figure 4c illustrates the nuclear displacements associated with the three key Franck–Condon active modes.

We note that these data also contain nonresonant signatures of impulsive stimulated Raman scattering of the methanol solvent. For example, in the ω_3_ = 14,770 cm^–1^ traces (Figure 4b), there is a solvent peak at 1040 cm^–1^. Its relative intensity is weak in comparison with the wavepackets from the Rhod700 solute signal.

#### 3.2.2 2DES Spectroscopy

**Figure 5:**
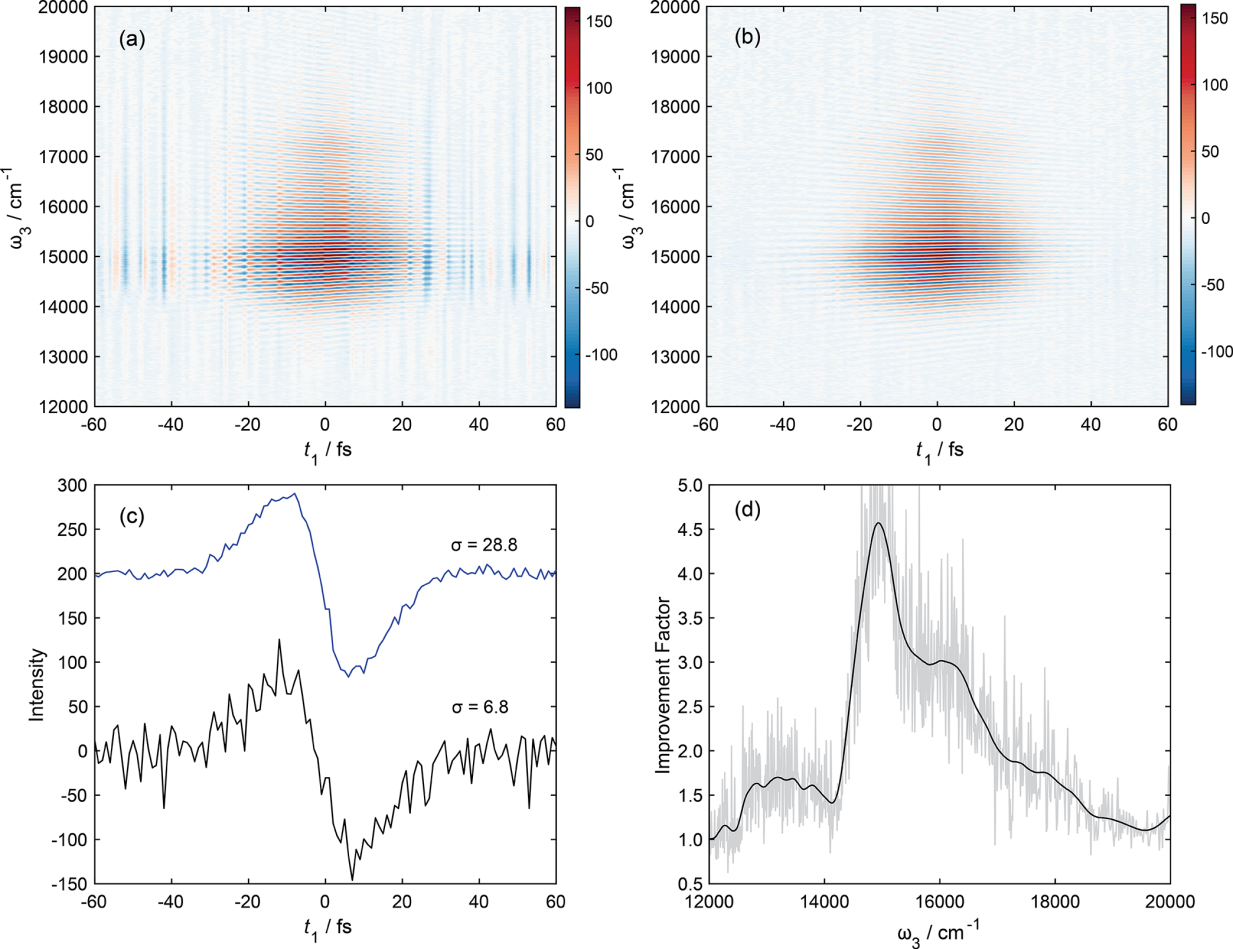
Raw 2DES(*t*_1_,ω_3_) interferograms for Rhod700 in methanol at *t*_2_ = 1000 fs (a) without and (b) with reference detection. Both 2D surfaces were acquired with 100 laser shots per *t*_1_ delay and only one spectral average. (c) Slices at ω_3_ = 14,925 cm^–1^ along *t*_1_–ω_3_ surfaces shown in panels a and b illustrate the improvement in the signal-to-noise ratio, σ (defined in the text) due to wavelength-dependent reference detection. (d) The probe-wavenumber dependent S/N ratio improvement factor determined from these data (gray line). A Gaussian smoothing function was applied to these data to reduce the inherent noise associated introduced from the unreferenced data (black line).

Raw 2DES interferograms of Rhod700 in methanol for a waiting time of 200 fs are shown in Figure 5a,b, without and with wavelength-dependent normalization, respectively. Each *t*_1_ delay in the 2DES(*t*_1_–ω_3_) surface was averaged for 100 laser shots. Comparing Figure 5a,b, it is immediately obvious that the signal-to-noise ratio is vastly improved for the data with reference detection. Like prior studies, we quantify the improvement in S/N in the time domain so that factors such as apodization do not impact the reported values.^85^ For an interferogram slice at a given ω_3_ (e.g., Figure 5c), we define the S/N ratio (σ) as the maximum signal intensity divided by the standard deviation of the baseline at long *t*_1_ delays where the signal has decayed to baseline. This analysis for ω_3_ = 14,925 cm^–1^ returns values of σ = 6.8 and 28.8 for unreferenced and referenced data, respectively, and an S/N improvement of 4.2 with reference detection at this specific probe wavenumber. The probe-wavenumber dependence to the S/N improvement for these data is shown in Figure 5d and demonstrates an improvement up to almost 5×. Similar values of σ were obtained for data recorded at all of the measured *t*_2_ delays investigated.

It is important to note that given that the duty cycle of our experiment is 1/4, the 2DES(*t*_1_–ω_3_) surface shown is constructed from only 25 shots per *t*_1_ delay that contain the necessary χ^3^ 2DES signal. The remaining 75 shots are required to remove unwanted scatter and pump–probe contributions. With the sampling of the *t*_1_ delay used (±60 fs in 1 fs steps), a full 2DES(*t*_1_–ω_3_) surface is acquired within 20 s, meaning after the surface is averaged three times, the total collection time is only 1 min per *t*_2_ time delay.

**Figure 6:**
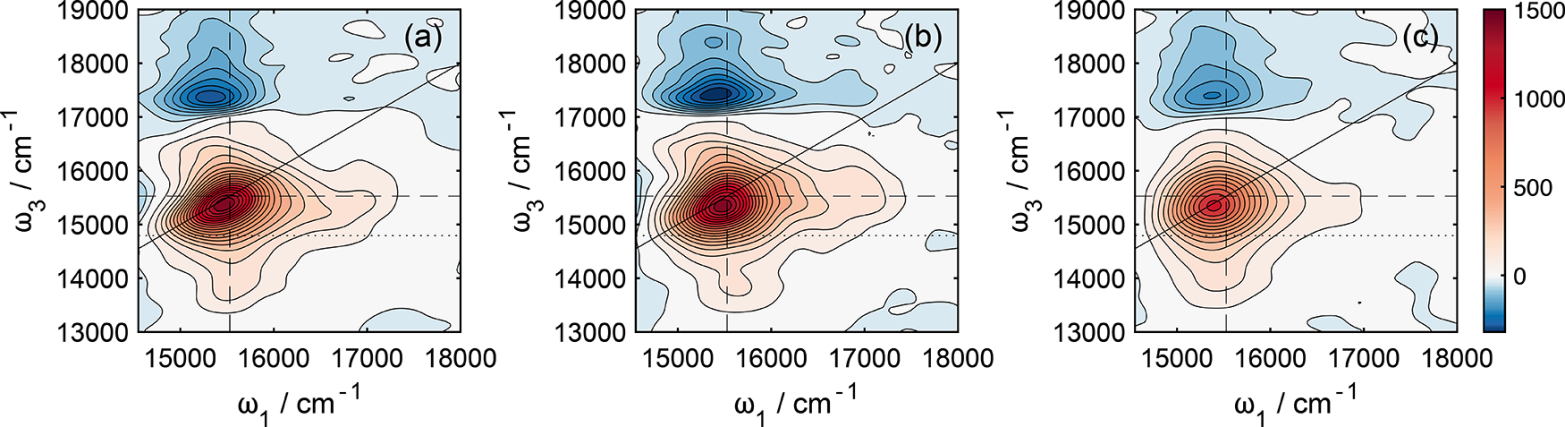
2DES spectra of Rhod700 at waiting times = (a) 104 fs, (b) 144, and (c) 1000 fs. All data were collected using wavelength-dependent reference detection. Data were acquired with 100 shots per *t*_1_ delay, and each 2DES surface was averaged for 3 cycles. Dashed lines on the probe axes correspond to the absorption and fluorescence maxima determined with steady-state spectroscopy.

Three example total (real) 2DES spectra acquired at different *t*_2_ waiting times are shown in Figure 6: at the earliest time delays (Figure 6a), the GSB/SE feature centered at ω_1_ = 15,500 cm^–1^ and ω_3_ = 15,800 cm^–1^ exhibits diagonal elongation. With increasing *t*_2_ delays (Figures 6b,c), the correlation between excitation and detection frequencies is lost, as expected through spectral diffusion.

As expected from the corresponding pump–probe data (Figure 3b), these 2DES spectra exhibit rich oscillations as a function of the waiting time. To isolate the vibrational wavepacket contribution to 2DES spectra, a similar procedure was taken as outlined in prior studies.^3,7,83,86^ The nonoscillatory population relaxation dynamics were removed from each point of the 2D ω_1_–ω_3_(*t*_2_) correlation maps by fitting data at each point to a spline function. A Fourier transform of the complex-valued wavepacket residuals, along the *t*_2_ delay, yields the coherence amplitude 2D beatmaps ω_1_–ω_3_(ω_2_).

**Figure 7:**
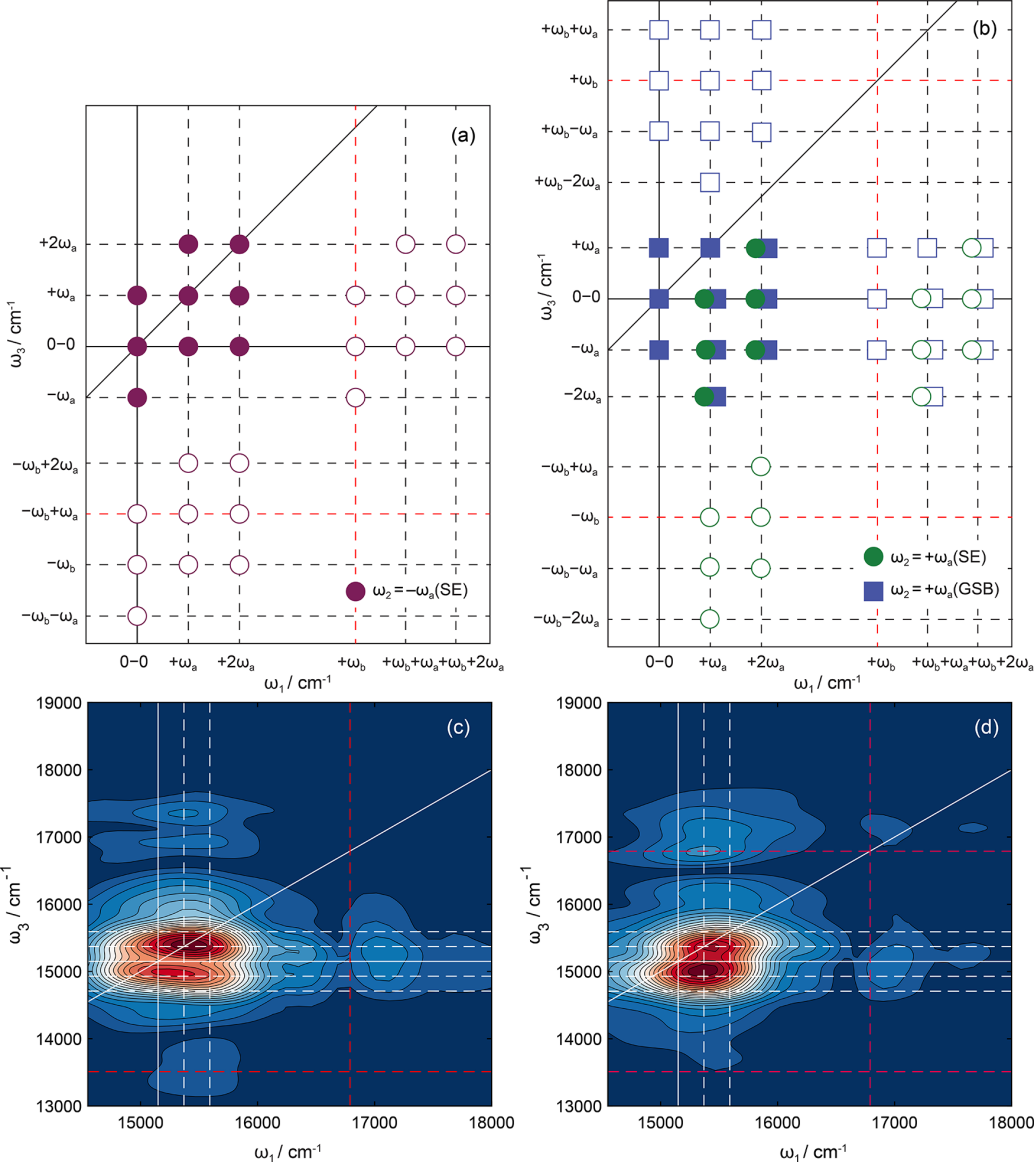
Theoretical beatmaps (rephasing pathways) for ω_2_ = (a) −ω_a_ and (b) +ω_a_ indicating the expected peak locations for vibronic transitions involving the fundamental mode, ν_a_, and a coupled mode, ν_b_, where the associated frequencies satisfy the ω_a_ < ω_b_ relation. The theoretical beatmaps were predicted using a combination of the double-sided Feynman diagrams illustrated in Figures S8–S11. Experimental rephasing coherence beatmaps for (c) −220 cm^–1^ and (d) +220 cm^–1^. The experimentally derived data are overlaid with solid white lines to indicate the S_1_–S_0_ 0–0 transition energy (15,150 cm^–1^). Horizontal and vertical dashed white lines indicate the energies associated with one or two quanta of the fundamental mode, ν_a_. Dashed red lines indicate the expected energies for the cluster of combination bands with a second vibrational mode ν_b_.

As the 2DES data and resulting beatmaps are complex-valued, we can distinguish between the positive and negative wavepacket frequencies. Since only specific vibrational wavepacket signal pathways, including a single mode, generate positive or negatively signed coherences, it is possible to determine whether the vibrational frequencies correspond to ground or excited state wavepackets. Using the convention previously determined,^33,80,83,86^ we define positive vibrational coherence signals as |*g*_0_⟩⟨*g*_1_| ∝ e^+*i*ω_01_*t*_2_^ and negative coherence signals thus as |*g*_1_⟩⟨*g*_0_| ∝ e^–*i*ω_01_*t*_2_^. Example beatmaps extracted from rephasing 2D signals are shown in Figure 7 for ω_2_ = ±220 cm^–1^, which is assigned to the out-of-plane mode, ν_160_—the associated nuclear displacements are shown in Figure 4c. These data are accompanied by theoretical beatmaps derived from double-sided Feynman diagrams (see Figures S8–S11) highlighting the expected locations where vibronic transitions associated with GSB and SE pathways will contribute to the 2DES spectra.^3,78,80,87^

The theoretical beatmaps are comprised of two varieties of vibronic transitions, and both oscillate at frequency ω_a_ in the *t*_2_ waiting time. The first set is those that involve vibronic transitions of only the fundamental vibrational mode, ν_a_, and are highlighted with solid symbols in Figure 7a,b. The physical origin of these signals has been well documented.^3,78,80,88^ The second set of transitions also oscillates at frequency ω_a_ in the *t*_2_ waiting time but involves signal pathways that include a combination of vibronic transitions of ν_a_ with a second mode, ν_b_—these transitions are labeled with open circles in the theoretical diagram. For such signals to arise, there must be vibronic coupling between the two different nuclear modes. This latter class of coupled vibronic transitions has only been reported in a few experimental and theoretical 2DES spectroscopic studies.^73,89,90^ Given the extremely broad bandwidth and short laser pulse durations used in our study, it is inevitable that we will impulsively drive many wavepackets associated with Franck–Condon active vibrations. Depending on the electronic structure of the molecule, some of these vibrations will be anharmonically coupled.

For vibronic transitions involving a single vibrational mode, excited state wavepackets will only contribute to negative frequency beatmaps (Figure 7a) in the form of SE signals (solid purple circles). Conversely, ground and excited state vibrational wavepackets of the same frequencies are predicted to contribute to positive frequency coherence maps (e.g., Figure 7b, see solid blue squares and solid green circles).

In both the experimental positive and negative frequency wavepacket maps associated with 220 cm^–1^, fundamental transitions involving solely ν_a_ (assigned to ν_160_) clustered around the S_1_–S_0_ origin (ω_1_ = ω_3_ = 15,150 cm^–1^, e.g., 0,0) dominate (e.g., Figure 7c). Given the strong signal in the negative frequency beatmap associated with 220 cm^–1^ (where SE, and thus the S_1_ state, wavepacket signals only contribute) and the “chair”-like pattern in the positive frequency map (Figure 7d), which also corresponds to SE signals, it is tempting to suggest that this frequency corresponds solely to an excited state wavepacket.

Despite bringing insights required to interpret experimental data and the expected locations at which different vibronic transitions should appear, the theoretical beatmaps do not give an indication of the expected relative intensities of the features in the experimentally derived spectra. For the two 220 cm^–1^ beatmaps, shown in Figure 7c,d, there is significantly greater intensity evident for features that appear at frequencies associated with one or more quanta of excitation in ν_a_. For example, with reference to the origin in Figure 7c at (ω_1_,ω_3_) = (0,0), the features located at (ω_1_,ω_3_) = (ω_a_,ω_a_) and (2ω_a_,ω_a_) have greater intensity than those located at the origin. This propensity for high vibrational excitation in ν_160_, associated with the 220 cm^–1^ frequency, indicates that there must be significant displacement along the associated vibrational potential in the S_1_ ← S_0_ electronic transition of Rhod700. This experimental finding is corroborated by the significant planar to nonplanar change in geometry after photoexcitation predicted by DFT and TDDFT calculations—see Figure S3.

The secondary sets of peaks located further away from the S_1_–S_0_ origin (open symbols in theoretical beatmaps) involve vibronic transitions with a secondary mode, ν_b_, that has a corresponding frequency of 1640 cm^–1^ and are assigned to ν_31_—an in-plane ring breathing mode (see Figure 4c). The Feynman diagrams (Figures S10 and S11) indicate that these coupled vibronic interactions occur during either the coherence or echo times on either the ground or excited potential energy surfaces. DFT and TDDFT calculations predict that the associated frequencies with ν_31_ differ by ∼70 cm^–1^ on the S_0_ (1643 cm^–1^) and S_1_ (1577 cm^–1^) electronic states (see Table S2). Clearly, the S_0_ calculated frequency more closely corresponds with peak spacing observed experimentally (1640 cm^–1^), implying that only the subset of transitions proposed theoretically involving this second mode contribute to these specific beat spectra, e.g., those where the vibrational coherences of ν_b_ (in *t*_1_ or *t*_3_) occur on the ground electronic state—in agreement with a 2DES study of molecular dyes by Schultz et al. where they concluded that it is not possible to simply ascribe all features in negative rephasing 2DES beatmaps to excited state wavepackets.^87^ However, given the predicted changes in Rhod700 nuclear geometry upon photoexcitation, it is not surprising that in-plane ring breathing modes are coupled to out-of-plane deformation motions, and these manifest in vibronic transitions.

We investigated whether these additional features involving a secondary vibrational mode, ν_b_, could be the result of “interferences” between harmonically coupled modes.^87^ One key manifestation of this effect is the observation of vibrational coherences in the *t*_2_ delay that beat at the difference frequency associated with the two modes: ω_a_–ω_b_ (or ω_b_–ω_a_). The Rhod700 2DES power spectrum, shown in Figure S7, does not show prominent signatures of these difference beat frequencies. Therefore, we conclude that for Rhod700, these coupled wavepacket signals arise from anharmonic coupling between two different vibronic modes, as per studies the conclusions of a study of oxazine 720.^73^

**Figure 8:**
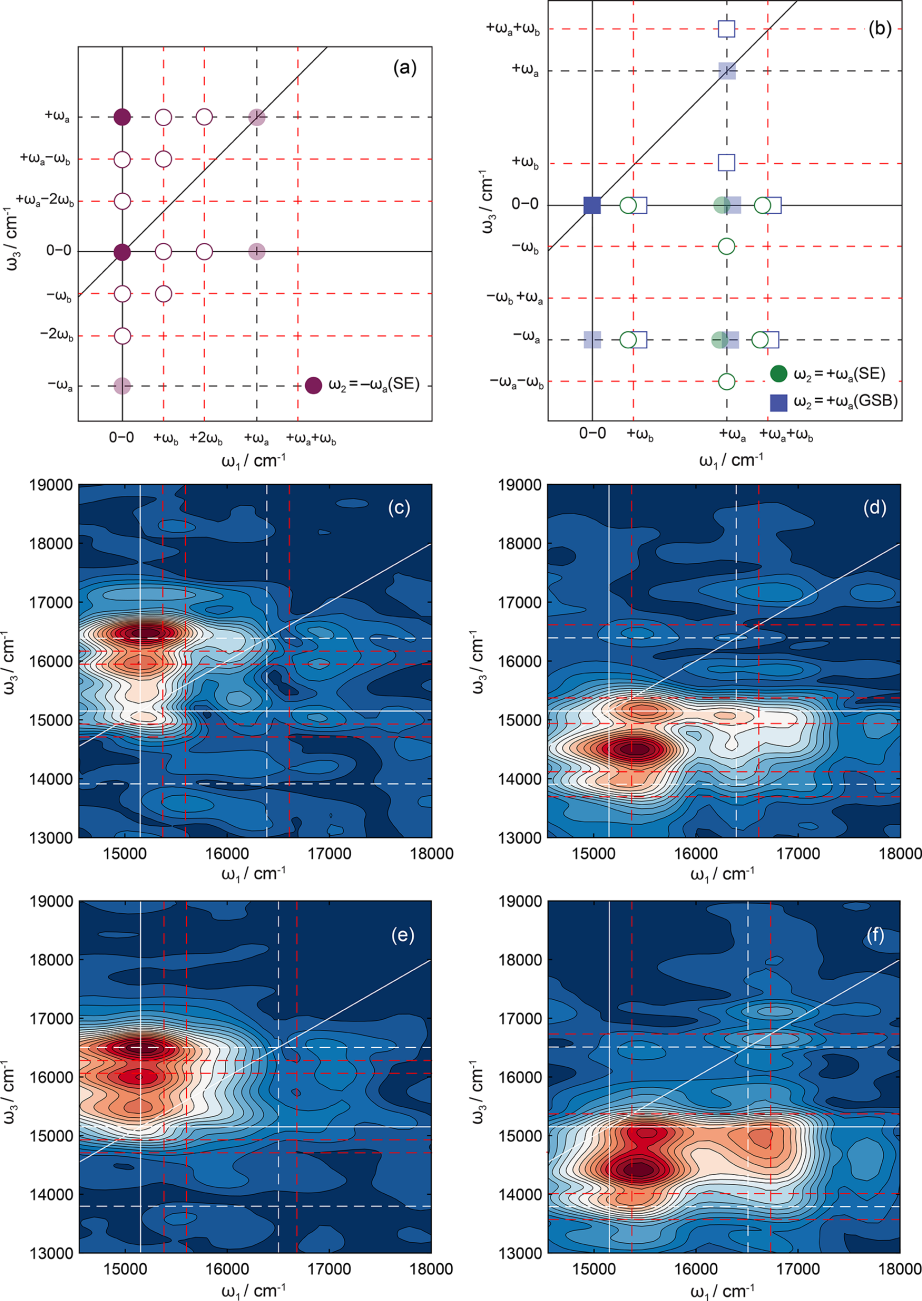
Theoretical beatmaps (rephasing pathways only) for ω_2_ = (a) −ω_a_ and (b) +ω_a_ indicating the expected peak locations for vibronic transitions involving the fundamental frequency, ω_a_, and a coupled mode, ω_b_, where ω_a_ > ω_b_. Beatmaps were predicted using double-sided Feynman diagrams shown in Figures S8–S11 and combinations thereof. Experimental rephasing coherence beatmaps for (c) −1240 cm^–1^, (d) +1240 cm^–1^, (e) −1360 cm^–1^, and (f) +1360 cm^–1^. Overlaid lines correspond to the same peak positions as detailed in the Figure 7 caption.

Figure 8 shows theoretical and experimental rephasing coherent beatmaps associated with two higher frequency wavepackets of 1240 and 1360 cm^–1^, corresponding to ν_72_ and ν_55_. These high-frequency modes are coupled to low-frequency vibrational modes, giving rise to different beatmap patterns; see Figure 8a,b. The nuclear motions associated with ν_72_ and ν_55_ involve in-plane ring breathing motions of Rhod700, e.g. Figure 4c (see Table S2 for a full description of the normal modes). Again, our theoretical beatmaps are able to predict all of the main peak locations in experimental data for vibronic transitions associated solely with the fundamental vibration (solid symbols in Figure 8a,b). However, there is less vibronic progression associated with these vibronic transitions compared to the 220 cm^–1^ (cf. Figure 7). This may arise due to less Franck–Condon activity in ν_72_ and ν_55_ compared to ν_31_, or the driving laser pulse duration is insufficiently short to create vibrational coherences involving two quanta of these modes.

In addition to the features within the beatmaps associated with fundamental vibronic transitions (e.g., those solely involving ν_a_), a secondary set of features is evident and can be assigned to coupled vibronic transitions involving a lower frequency mode, ν_159_, associated with out-of-plane deformations, with frequency = 250 cm^–1^. From the evident patterns in the experimentally derived beatmaps and close correspondence with the theoretically predicted peak positions, there is far greater vibronic excitation in the low-frequency mode than in the high-frequency fundamental vibration.

**Figure 9:**
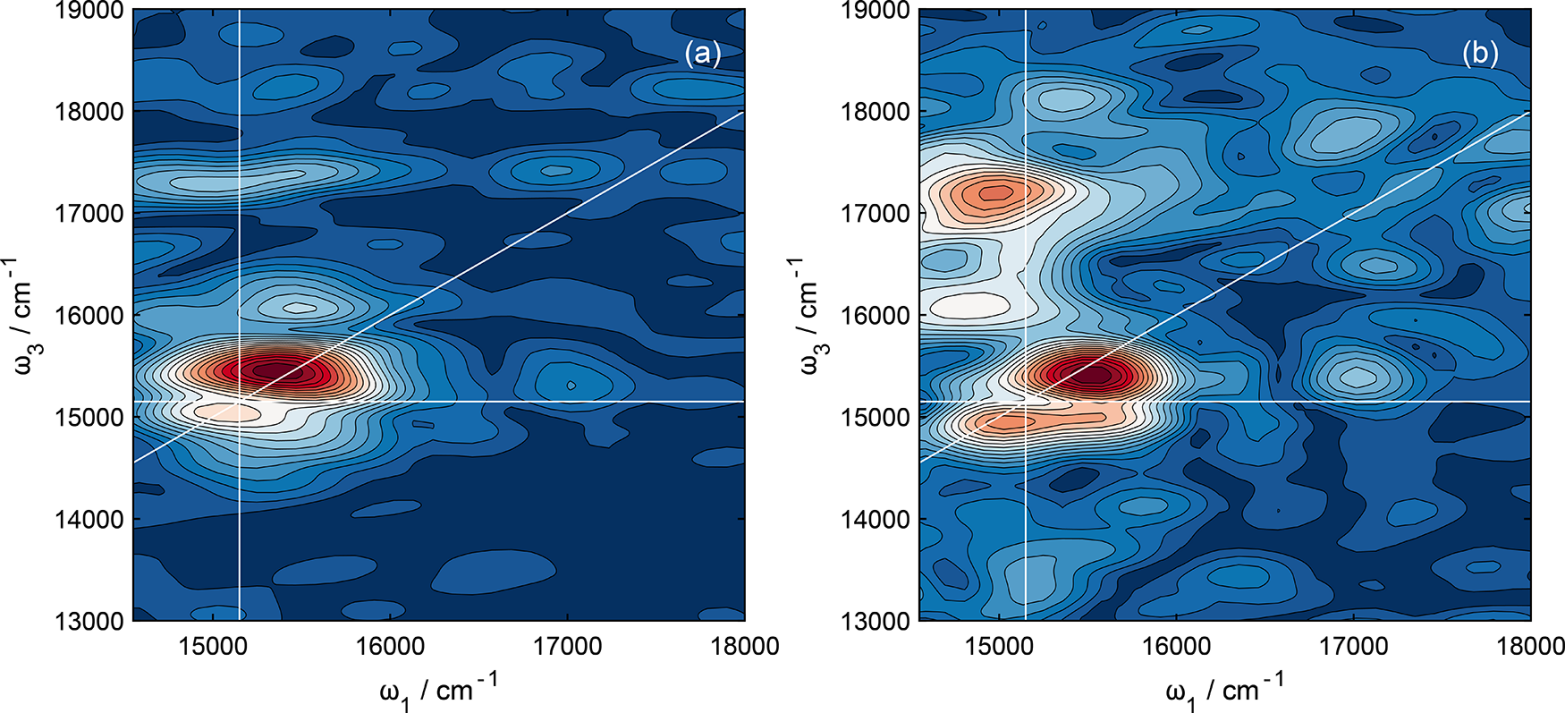
Comparison of +220 cm^–1^ rephasing beatmaps acquired for Rhod700 nm solutions with OD = 0.1 at 645 nm. Data shown in panel a was acquired with wavelength-dependent reference detection, whereas panel b did not correct for any fluctuations in the laser intensity. All data were acquired for the same number of shots and averages (100 shots per measurement, 3 averages per 2DES map).

To further exemplify how wavelength-resolved reference detection enhances the sensitivity of 2DES, we extended our studies to investigate Rhod700 samples with lower absorbance and how this impacts the quality of the 2D beatmaps. The oscillatory beatmaps are very sensitive to laser stability, and obtaining a high signal-to-noise in these data requires consistency to be maintained while scanning both *t*_1_ and *t*_2_ delays, an acquisition process that can take up to a period of an hour. Figure 9 gives an example rephasing beatmap associated with +220 cm^–1^ for data acquired at low concentrations (0.1 OD, ∼55 μM) of Rhod700 in methanol. It is evident that the signal-to-noise ratio of the referenced data is far superior to data acquired without reference detection, with the latter set clearly containing many artifacts. The data shown in Figure 9a show a close resemblance to data acquired at 3× higher concentrations; see Figure 7d. This example serves to emphasize the enhanced signal-to-noise afforded by wavelength-dependent reference detection for the 2DES ω_1_–ω_3_(*t*_2_) data sets from which the coherent beatmaps derive.

## 4 Conclusions

Using the 8 fs compressed ultrabroadband output from a hollow-core fiber laser source for a 2DES interferometer comprised solely of conventional optics, we have investigated the vibronic coupling of Rhodamine 700 in methanol solution. We have demonstrated, for the first time, the implementation of full-wavelength reference detection for *degenerate* 2DES using a ultrabroadband laser source. Our 2DES interferometer yields data with 4–5× higher signal-to-noise ratios compared to data collected without correcting for wavelength-dependent laser fluctuations. These data, despite using a 1 kHz rate Ti:sapphire laser and controlling the interpulse time delays with mechanical delay stages, yield fully spectrally averaged 2DES data within 1 min of data collection, and a S/N ratio of 28. The signal-to-noise enhancements are sufficiently large that we can collect good-quality data on samples with lower than typical optical densities at the peak absorption wavelength, 0.1 in a 200 μm path length flow cell, equating to concentrations of ∼55 μM. These advancements to improve the sensitivity of 2DES will remove the often-stringent demands of highly concentrated precious samples while maintaining the integrity of data. Due to the excellent signal-to-noise, ultrabroad bandwidth, and high temporal resolution, our 2DES study of Rhodamine 700 in methanol has revealed a variety of different vibronically coupled modes through anharmonic coupling, which we anticipate will be far more common than hitherto reported, except for a few notable examples in the literature.^73,90^

Our wavelength-dependent intensity normalization using a reference matrix for degenerate 2DES with visible/near-IR wavelengths builds on earlier pioneering mid-infrared 2DIR studies.^68,69^ It is expected that the same methodology will find utility in multidimensional spectroscopic methods that span other parts of the electromagnetic spectrum. For example, the recent surge of activity in generating tunable broadband deep-UV laser sources^91−93^ and phase-locked pulse-pair generation in the UV^94,95^ means wavelength-dependent normalization with a correlation matrix will help to improve data S/N ratios for 2DUV spectroscopy. We predict that the method should also enhance data collection with 2-color multidimensional spectroscopic methods such as 2DEV^96^ and 2DVE^97^ spectroscopies, which use extremely different pump and probe wavelengths. The implementation will require two different reference detectors, and we envisage that the correlation matrix will appropriately compensate for the different laser fluctuations, which will contribute to the overall nonlinear signals.

We envisage two further enhancements to improve the speed of data acquisition and thus the sensitivity of 2DES experiments with ultrabroad bandwidth acquired with a traditional boxcars geometry experiment: (i) Coupling wavelength-dependent reference detection method to rapid scanning of the *t*_1_ coherence time. The latter innovation has been recently demonstrated by the groups of Tiwari^49^ and Chen^98^ for 2DES spectroscopy, yielding multifold enhancements in signal-to-noise ratios. (ii) High-repetition rate lasers, up to 100 kHz, are becoming more commonplace within the field of multidimensional optical spectroscopies,^48,49,99^ and yield 2D spectra with outstanding signal-to-noise ratios. However, due to the longer fundamental pulse durations of these laser systems, this often comes with the penalty of reduced associated bandwidths. Recent advancements in hollow-core fiber technology indicate that ultrabroadband continua with high peak pulse energies can be derived from these longer pulse duration laser sources at high-repetition rates,^100^ and will likely be at the next forefront of 2DES optical spectroscopy method development, heralding yet further advances in experimental sensitivity.

## Data Availability

The data underlying this study are openly available at Zenedo at doi: 10.5281/zenodo.14894853.

## Supporting Information Available

Additional pulse characterization; calculated vertical excitation energies; S_0_ and S_1_ Rhod700 minimum energy geometries; normal mode assignments; full steady-state absorption spectrum; comparison of pump–probe spectroscopy with and without phase modulation; examples of phased 2DES data; coherent beatmap power spectra; and double-sided Feynman diagrams associated with coherent beatmaps (PDF)
