# Supplementary material for: Two-Dimensional Electronic Spectroscopy of Rhodamine 700 Using an 8 fs Ultrabroadband Laser Source and Full-Wavelength Reference Detection
Source: J Phys Chem A. 2025 Mar 5;129(15):3537–51. doi: 10.1021/acs.jpca.4c08494 (PMC12010339; doi:10.1021/acs.jpca.4c08494)
Supplement: Supplementary file 1 — jp4c08494_si_001.pdf [file jp4c08494_si_001.pdf]

Supporting Information for:

Two-Dimensional Electronic Spectroscopy of Rhodamine 700 using an 8-fs Ultrabroadband Laser Source and Full Wavelength Reference Detection

Camilla Gajo,[‡] Caleb J.C. Jordan[‡] and Thomas A.A. Oliver*

School of Chemistry, University of Bristol, Bristol, BS8 1TS, UK

[‡]These authors contributed equally to this work

*Author for correspondence: tom.oliver@bristol.ac.uk

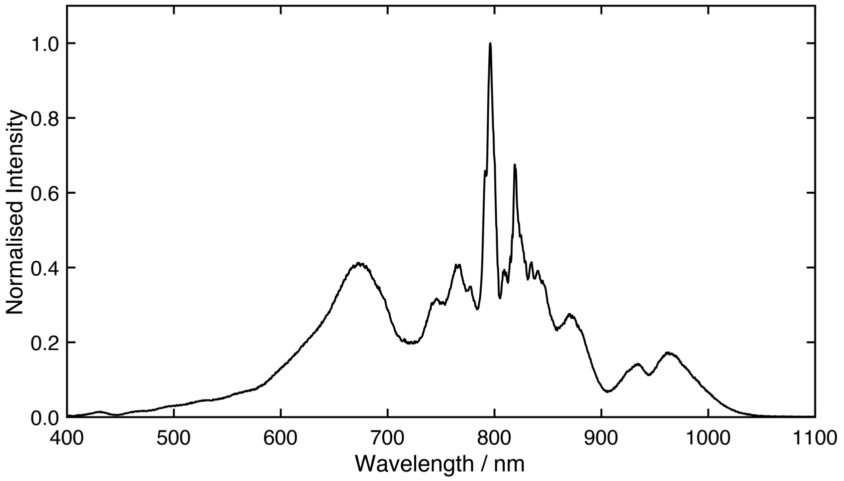

Figure S1. Spectrum of the hollow core fibre laser source.

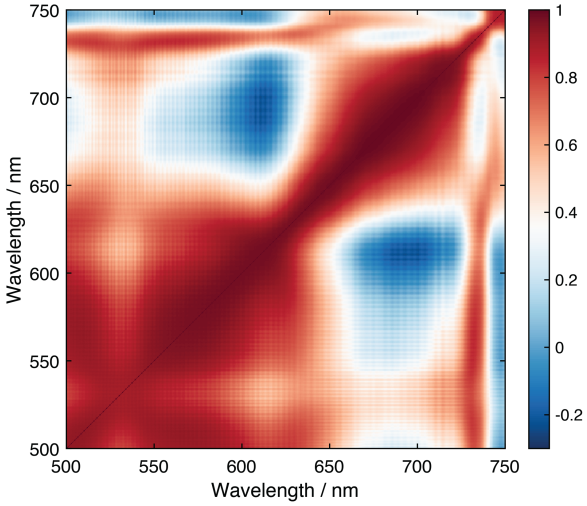

Figure S2. Spectral correlation map for our laser source, with coefficients ranging from –0.3 (blue) to +1 (red). These data show a strong correlation (as expected) along the diagonal, and positive correlations for near-neighbouring wavelengths. However, there are also wavelength regions which are uncorrelated or anti-correlated. The above plot was generated by taking vectors of points corresponding to the $\Delta T/T$ values for each pixel in the absence of true signal (such that the $\Delta T/T$ value effectively represented the fluctuation from the mean), and calculating the Pearson's linear correlation coefficient between all permutations of vector pairs. The modulation near 725 nm likely arises from some self-phase modulation with the short-wavelength tail of the 800 nm driving pulse.

Table S1. Calculated vertical and adiabatic energies of the S_1 and S_2 electronic states of Rhodamine 700 using TDDFT/TDA/ωB97XD/def2-SVP with a methanol EIFPCM.

Electronic State	Vertical Excitation Energy / eV	Oscillator Strength (*f*)	Adiabatic Excitation Energy/ eV
S_1	2.73	1.410	2.38
S_2	3.23	0.001	—

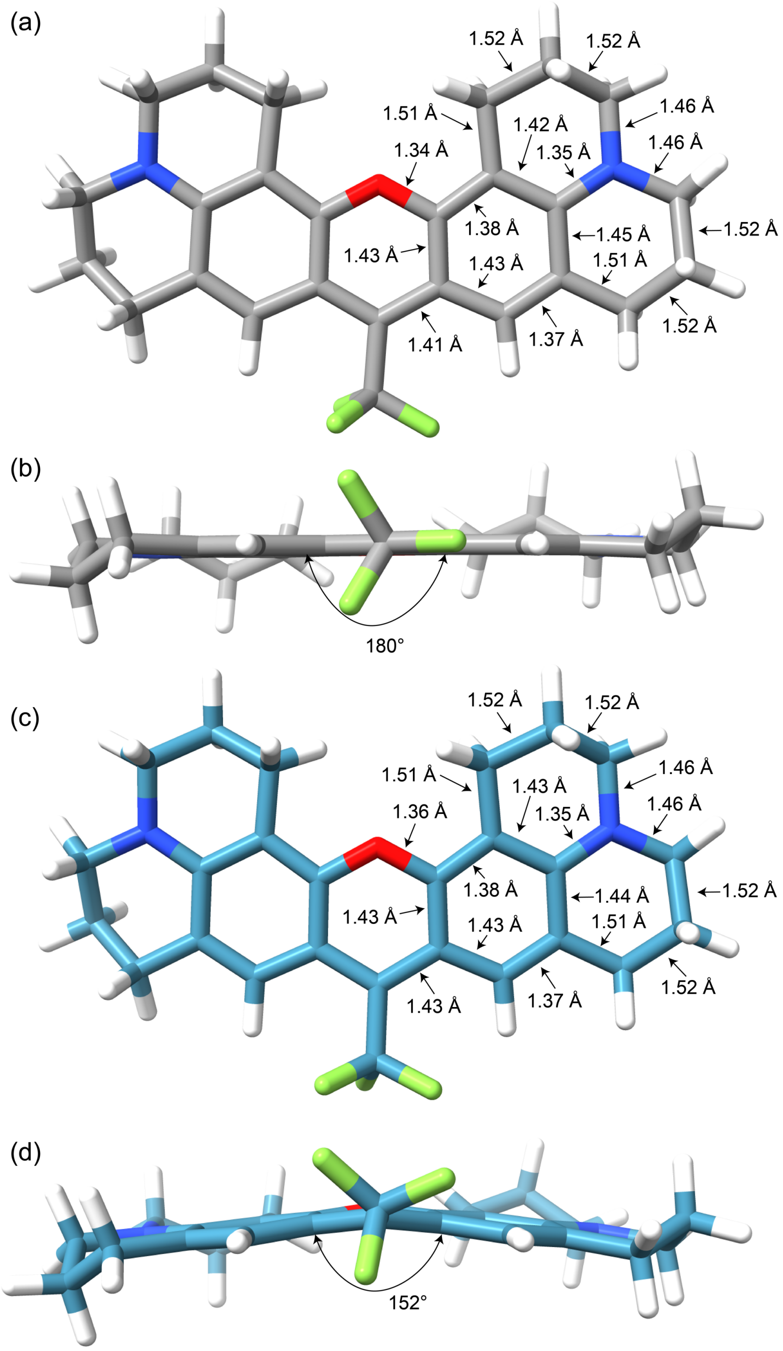

Figure S3. Minimum energy S_0 and S_1 state Rhod700 structures calculated at the (TD)DFT/TDA/ωB97XD/def2-SVP with a methanol EIFPCM solvent model. Overlaid are labelled bond lengths only for half the molecule as Rhod700 exhibits pseudo-C_2 rotation symmetry. The side view illustrates the level of planarity within the molecule.

Table S2. Vibrational wavepacket assignments based on harmonic (TD)DFT/TDA/ωB97XD/def2-SVP normal mode frequency calculations. Assignments are based on similarity with calculated frequency and geometric changes. Normal modes are labelled using Herzberg notation[1] based on the ordering in the S_1 electronic state.

Mode	Observed Freq / cm^{-1}	Calculated Freq / cm^{-1}	Assignment
ν_{31}	1650	*1643 (S_0)<br>1577 (S_1)	In-plane xanthene antisymmetric ring-breathing mode
ν_{47}	1500	1523 (S_0)<br>*1473 (S_1)	In-plane symmetric pyran C–C–C stretching mode
ν_{55}	1360	1405 (S_0)<br>1398 (S_1)	Symmetric ring breathing mode centred on pyran ring
ν_{72}	1240	1259 (S_0)<br>1266 (S_1)	Symmetric xanthene ring breathing mode
ν_{159}	250	266 (S_0)<br>256 (S_1)	Pyran ring breathing stretch, coupled to out-of-plane symmetric butterfly motion of quinolizidine rings
ν_{160}	223	162 (S_0)<br>*242 (S_1)	Pyran ring breathing stretch, coupled to out-of-plane anti-symmetric butterfly motion of quinolizidine rings

*Denotes preferred assignment based on either closet correspondence with experimentally observed frequency, or analysis of 2DES beatmaps.

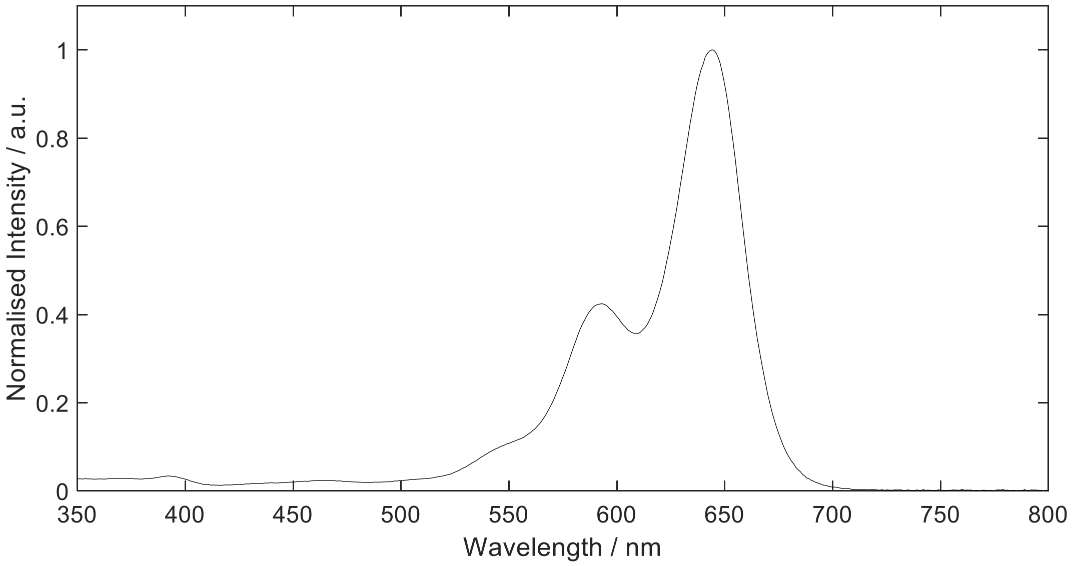

Figure S4. Normalised UV-visible absorption spectrum of Rhodamine 700 in methanol between 350 and 800 nm.

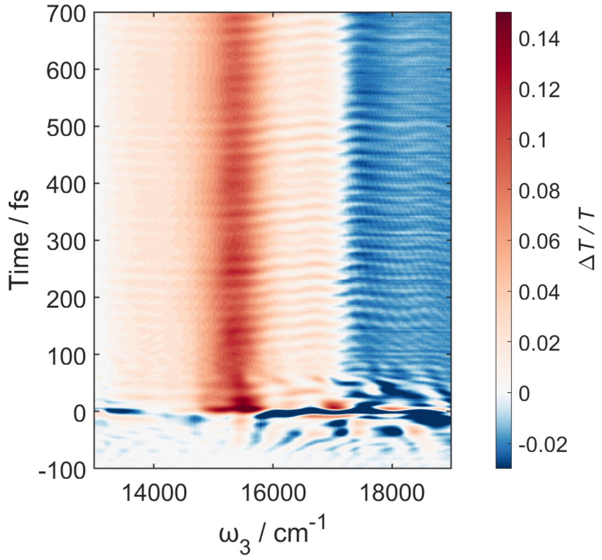

Figure S5. Comparison of degenerate broadband pump-probe spectra of Rhodamine 700 in methanol obtained (a) without and (b) with modulation of the t_2 delay by λ/2.

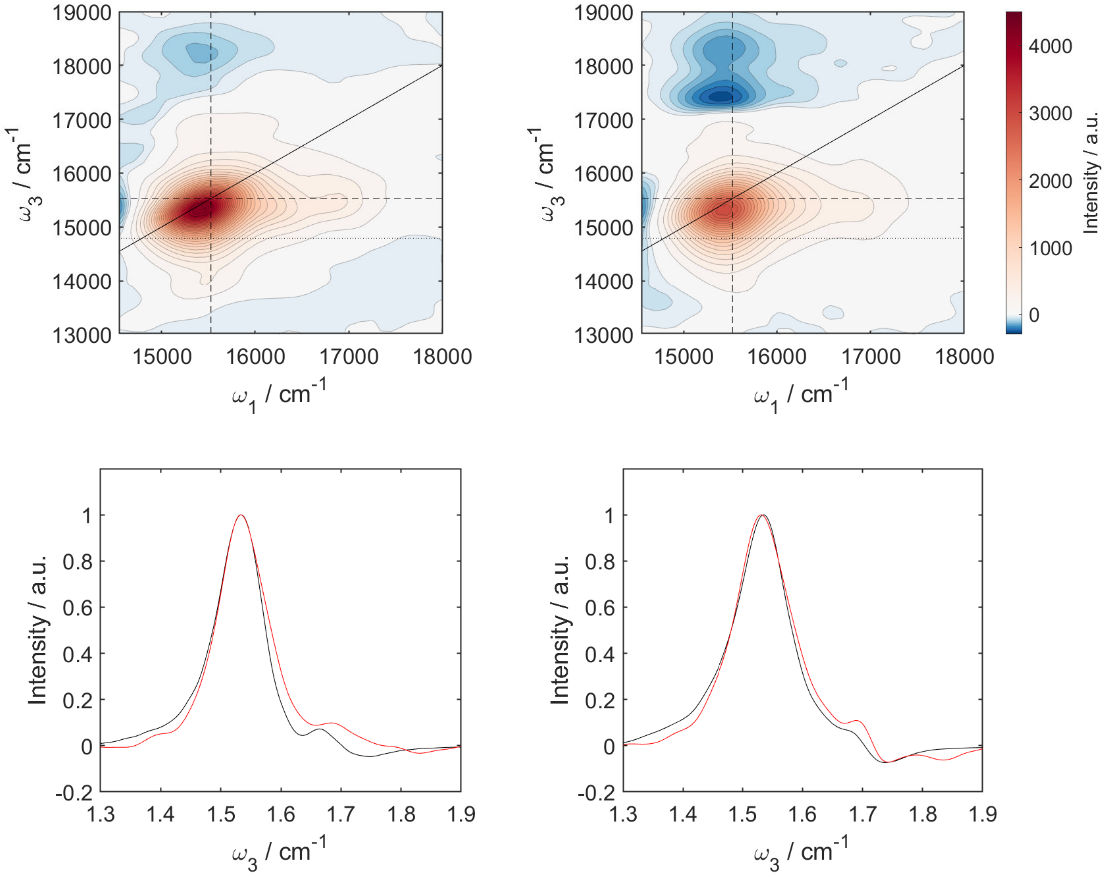

Figure S6. Two examples of 2DES spectra and projection slice fits. (a) 2DES map for t_2 = 96 fs and (b) 2DES map for t_2 = 696 fs. (c) Smoothed pump-probe signal at t_2 = 96 fs (black line) and 2DES map projection obtained by integration over ω_1 (red line), and (d) the same for t_2 = 696 fs.

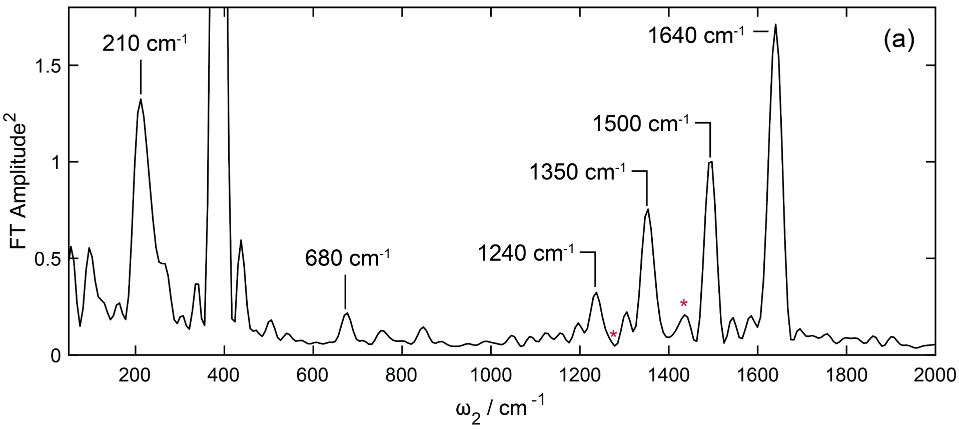

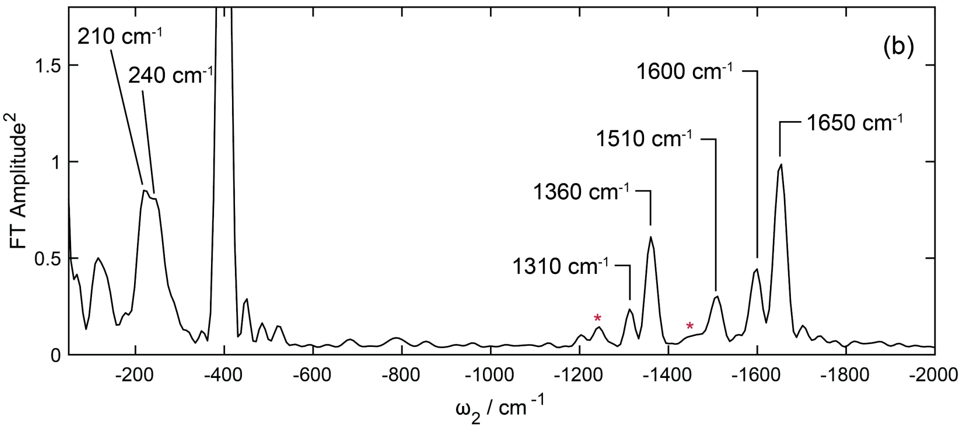

Figure S7. Coherent beat map power spectra of Rhod700 in methanol for (a) positive and (b) negative frequencies. Peaks denoted with * indicate where interferences between two different wavepackets would be expected based on the study by Schultz *et al.*.[2]

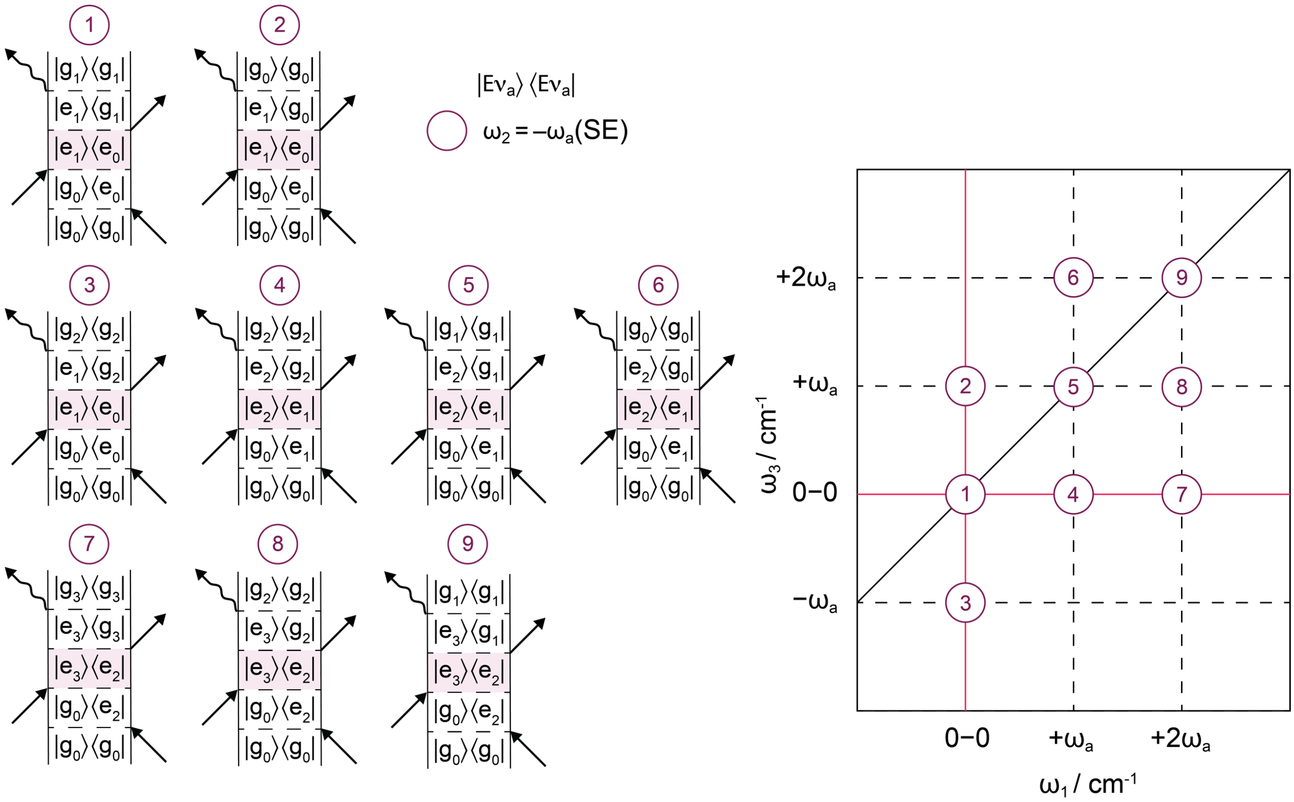

Figure S8. Double-sided Feynman diagrams for the rephasing pathways associated with coherent wavepackets of a single vibrational mode, ν_a, (negative frequencies in ω_2).[3–6] The ket and bra are labelled with respect to the electronic state E, corresponding to either the ground or excited state, g (S_0) or e (S_1), respectively, with the subscript denoting the number of vibrational quanta in mode ν_a. The theoretical beatmap illustrates the anticipated locations of the associated signals which oscillate at frequency ω_a.

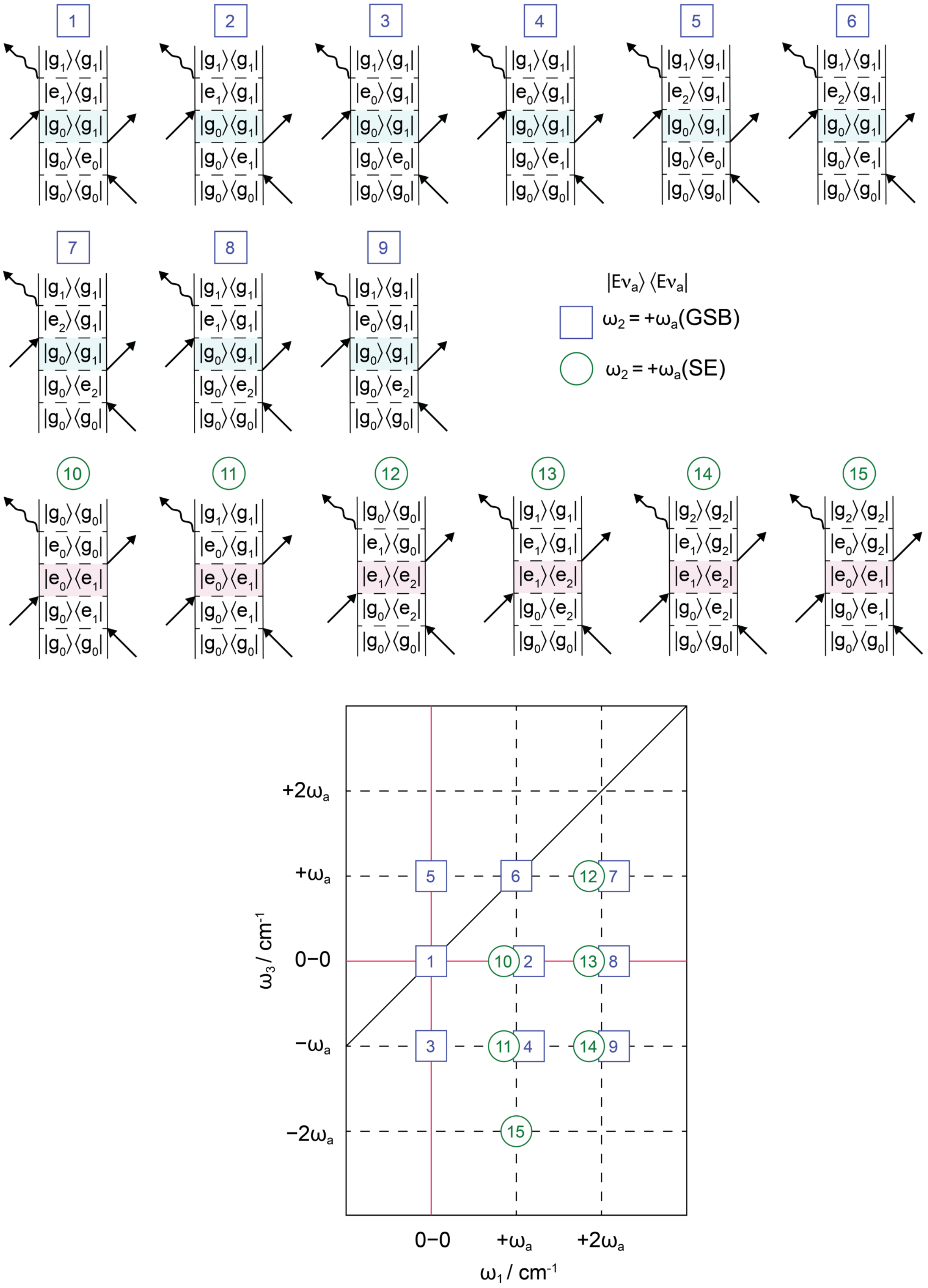

Figure S9. Double-sided Feynman diagrams for the rephasing pathways associated with coherent wavepackets of a single vibrational mode, ν_a, (positive frequencies in ω_2).[3–6] The theoretical 2D beatmap illustrates the location of associated signals which oscillate at frequency ω_a.

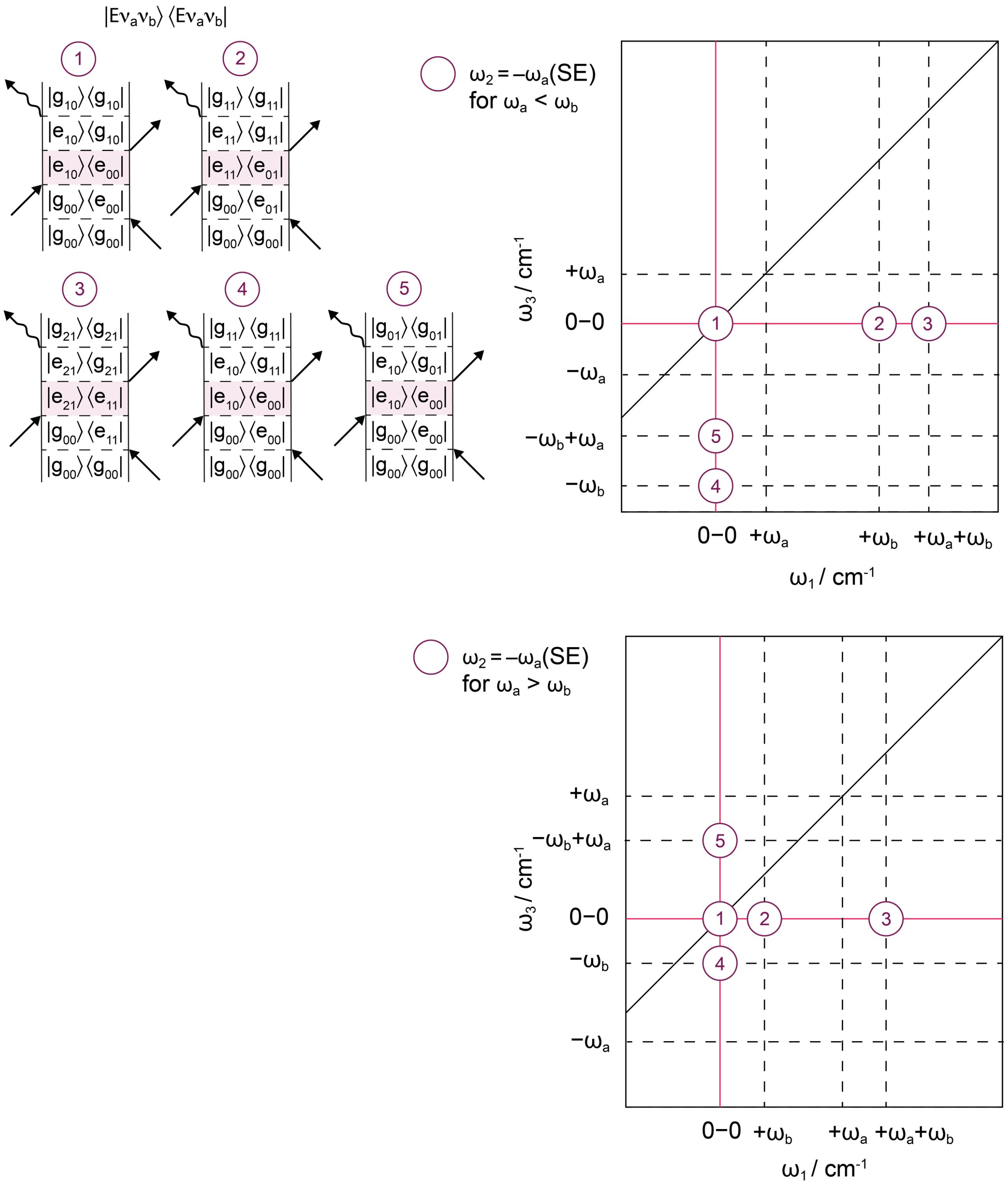

Figure S10. Double-sided Feynman diagrams for the rephasing pathways associated with coherent wavepackets of two coupled vibrational modes, ν_a and ν_b, for beat frequency = $-\omega_a$. The ket and bra are labelled with respect to the electronic state E, corresponding to either the ground or excited state, g (S_0) or e (S_1), respectively, with the subscripts denoting the number of vibrational quanta in mode ω_a and ω_b. The theoretical beat map illustrates the anticipated locations of the associated signals, for the case of $\omega_a < \omega_b$ and $\omega_a > \omega_b$. The oscillatory signal pathways shown are not definitive, and many signals involving vibronic transitions including only ν_a have been omitted for clarity.

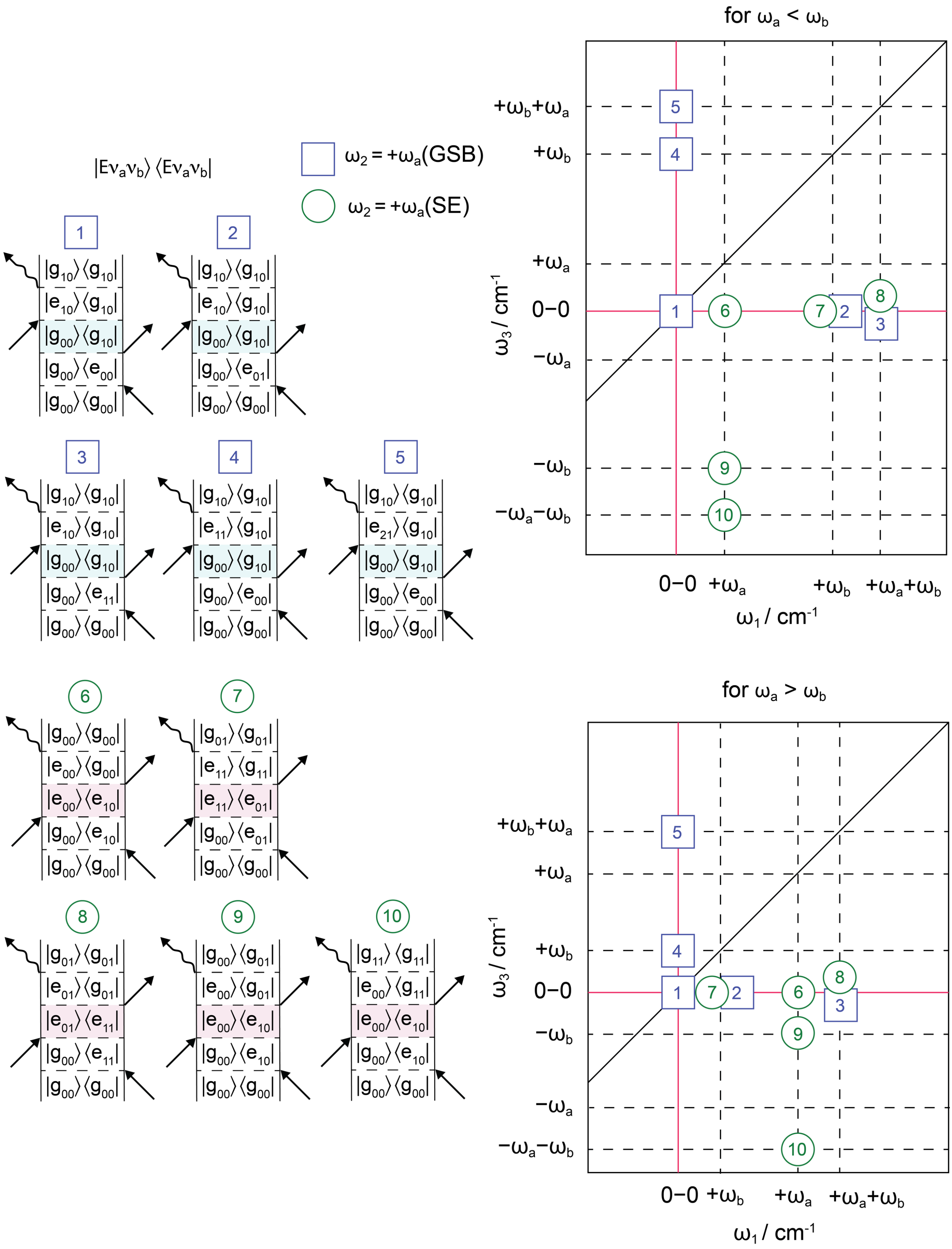

Figure S11. Double-sided Feynman diagrams for the rephasing pathways associated with coherent wavepackets of two coupled vibrational modes, ν_a and ν_b at beat frequency = $+\omega_a$. The theoretical beat map illustrates the some of the major coupled vibronic transitions that will contribute to the experimental spectra for the case of $\omega_a < \omega_b$ and $\omega_a > \omega_b$. The oscillatory signal pathways shown are not definitive, and the majority of vibronic signals involving solely ν_a are omitted for clarity.

References

(1) Herzberg, G. *Infrared and Raman Spectra of Polyatomic Molecules*; Van Nostrand: Princeton, 1945.

(2) Schultz, J. D.; Kim, T.; O'Connor, J. P.; Young, R. M.; Wasielewski, M. R. Coupling between Harmonic Vibrations Influences Quantum Beating Signatures in Two-Dimensional Electronic Spectra. *J. Phys. Chem. C* **2020**, *126* (1), 120–131. https://doi.org/10.1021/acs.jpcc.1c09432.

(3) Butkus, V.; Zigmantas, D.; Valkunas, L.; Abramavicius, D. Vibrational vs. Electronic Coherences in 2D Spectrum of Molecular Systems. *Chem. Phys. Lett.* **2012**, *545*, 40–43. https://doi.org/10.1016/j.cplett.2012.07.014.

(4) Seibt, J.; Pullerits, T. Beating Signals in 2D Spectroscopy: Electronic or Nuclear Coherences? Application to a Quantum Dot Model System. *J. Phys. Chem. C* **2013**, *117* (36), 18728–18737. https://doi.org/10.1021/jp406103m.

(5) Dean, J. C.; Rafiq, S.; Oblinsky, D. G.; Cassette, E.; Jumper, C. C.; Scholes, G. D. Broadband Transient Absorption and Two-Dimensional Electronic Spectroscopy of Methylene Blue. *J. Phys. Chem. A* **2015**, *119* (34), 9098–9108. https://doi.org/10.1021/acs.jpca.5b06126.

(6) Green, D.; Camargo, F. V. A.; Heisler, I. A.; Dijkstra, A. G.; Jones, G. A. Spectral Filtering as a Tool for Two-Dimensional Spectroscopy: A Theoretical Model. *J. Phys. Chem. A* **2018**, *122* (30), 6206–6213. https://doi.org/10.1021/acs.jpca.8b03339.
